# *Acinetobacter baumannii* ATCC 17978 encodes a microcin system with antimicrobial properties for contact-independent competition

**DOI:** 10.1099/mic.0.001346

**Published:** 2023-06-07

**Authors:** Fabiana Bisaro, Howard A. Shuman, Mario F. Feldman, Michael J. Gebhardt, Stefan Pukatzki

**Affiliations:** ^1^​ Department of Biology, The City College, City University of New York, New York, NY 10031, USA; ^2^​ Department of Molecular Microbiology, Washington University School of Medicine in St. Louis, St. Louis MO 63110, USA; ^3^​ Department of Microbiology, The University of Chicago, Chicago, IL 60637, USA; ^4^​ Department of Microbiology and Immunology, University of Iowa, Iowa City, Iowa 52242, USA; ^†^​ Present address: P.O. Box 1088, Sheffield, MA 01257, USA

**Keywords:** Acinetobacter baumannii, Fiu, Iron, Iron uptake receptor, Microcin, PiuA

## Abstract

*
Acinetobacter baumannii* is a multidrug-resistant opportunistic pathogen that persists in the hospital environment and causes various clinical infections, primarily affecting immunocompromised patients. *A. baumannii* has evolved a wide range of mechanisms to compete with neighbouring bacteria. One such competition strategy depends on small secreted peptides called microcins, which exert antimicrobial effects in a contact-independent manner. Here, we report that *A. baumannii* ATCC 17978 (AB17978) encodes the class II microcin 17 978 (Mcc17978) with antimicrobial activity against closely related *Acinetobacter *, and surprisingly, also *Escherichia coli* strains. We identified the genetic locus encoding the Mcc17978 system in AB17978. Using classical bacterial genetic approaches, we determined that the molecular receptor of Mcc17978 in *E. coli* is the iron-catecholate transporter Fiu, and in *Acinetobacter* is Fiu’s homolog, PiuA. In bacteria, the Ferric uptake regulator (Fur) positively regulates siderophore systems and microcin systems under iron-deprived environments. We found that the Mcc17978 system is upregulated under low-iron conditions commonly found in the host environment and identified a putative Fur binding site upstream of the *mcc17978* gene. When we tested the antimicrobial activity of Mcc17978 under different levels of iron availability, we observed that low iron levels not only triggered transcriptional induction of the microcin, but also led to enhanced microcin activity. Taken together, our findings suggest that *A. baumannii* may utilize microcins to compete with other microbes for resources during infection.

## Introduction

*Acinetobacter baumannii* is a nosocomial, opportunistic pathogen that causes a broad range of infections, including pneumonia, bacteremia, urinary tract infections, wound infections, meningitis, and sepsis. Although these infections mainly affect immunocompromised and critically ill patients [1–5], *A. baumannii* causes community-acquired infections with increasing frequency [1, 2]. Additionally, *A. baumannii* has been deemed a top global health threat due to high rates of multi-drug resistance and ability to survive various stresses such as desiccation [3] and disinfection [4]. Importantly, *A. baumannii* is often associated with polymicrobial infections and competes with other bacteria at the infection site for limited resources [5, 6].

In polymicrobial communities, bacteria compete for nutrients and space using contact-dependent strategies, including the type VI secretion system (T6SS), and contact-independent strategies, such as diffusible compounds (i.e. microcins) [7]. Multiple *A *. *baumannii* strains employ T6SSs against Gram-negative and Gram-positive competitors [8], whereby an attacking cell delivers toxic effectors to a prey cell via an elaborate nanomachine [9–12].

We reported that *A. baumannii* ATCC 17978 (AB17978) can kill *Escherichia coli* K12 in a T6SS-independent manner [11]. In contact-independent competition, some Gram-negative bacteria use microcins – small peptides (<10 kDa) that diffuse into the extracellular milieu – to antagonize neighbouring bacteria. Microcins are classified into class I microcins and class II microcins. Class I microcins are generally encoded on a plasmid and contain post-translational modifications. The class II microcins, which can be encoded either on a plasmid or in the chromosome and may contain minimal, if any, post-translational modifications [13], are widely spread across members of the *Enterobacterales* order [14] and the genetic loci encoding class II microcins often also include both immunity and self-transport systems [15]. Class II microcins can be further divided into either type IIa or type IIb depending on the propensity of the mature peptides to form disulphide bonds without post-translational modification (IIa) or exhibit siderophore-like activity (IIb) [16]. Examples of class IIa microcins include microcin V [17], microcin L [18], microcin N [19], microcin PDI [20] and microcin S [21]. All these class IIa microcins are produced by *E. coli *. Examples of class IIb microcins include the bactericidal microcin E492 (MccE492) produced by *Klebsiella pneumoniae* and microcin H47 (MccH47) produced by *E. coli* [22–25]. Class II microcin transport is mediated by the inner membrane protein Peptidase-containing ABC transporter (PCAT) and the outer membrane protein TolC. Using its peptidase domain, PCAT recognizes and cleaves the leader peptide to produce the mature form of the microcin, which is subsequently transported through the PCAT into the periplasm, where TolC mediates its export across the outer membrane into the extracellular milieu [15, 26].

Class II microcins interact with outer membrane proteins in the target bacterial cell, including iron uptake receptors and porins. While class IIa microcins interact with Cir, FepA, OmpF and Fiu, class IIb microcins interact with Fiu, FepA, Cir and IroN [27, 28]. Fiu is an iron-catecholate transporter that transports ferric iron bound to catecholate [29–31]. Following transport across the outer membrane, most class II microcins disrupt inner membrane potential to inhibit the growth of the target cell. MccE492 stably associates with the mannose permease transporter and leads to the formation of a pore and the eventual depolarization of the inner membrane [32–35]. In contrast, MccH47 kills competitor cells by targeting the proton channel of the ATP synthase complex, which also leads to depolarization of the target-cell inner membrane [23, 36]. As noted above, class II microcin-producing cells also employ self-immunity mechanisms to prevent self-intoxication. Little is known about these immunity strategies; in certain cases, protein complexes in the inner membrane specifically bind the microcin and interfere with the interaction between the microcin and its target [15, 34, 37, 38].

Microcin expression is often linked to iron availability and is regulated by the Ferric uptake regulator Fur [39, 40]. When iron is abundant, the Fur protein binds to specific DNA sequence motifs termed ‘Fur boxes’ [41, 42], leading to repression of iron-responsive genes. When iron levels are low, Fur binding diminishes, and the iron-responsive genes are expressed [39, 43]. Bacterial pathogens utilize a myriad of iron-acquisition systems as virulence factors to facilitate survival in iron-limiting conditions in their hosts [44–48]. Indeed, *A. baumannii* has evolved numerous iron-capturing mechanisms, including three Fur-regulated siderophore clusters that encode transporters for acinetobactin [49], baumannoferrin [50], and fimsbactin [48, 51]. Recently, the siderophore cluster encoding the machinery for the synthesis and uptake of fimsbactin was found in the mobile genetic element Tn6552 in AB17978, which also encodes a microcin cluster [51].

AB17978 was the first *A. baumannii* strain to be sequenced [52] and the first found to have an active T6SS encoded on the chromosome [11]. The T6SS is repressed by multidrug-resistance (MDR) plasmid pAB3, which encodes T6SS repressors, rendering AB17978 in a biphasic state: T6SS-negative and antibiotic-resistant, or T6SS-positive and antibiotic sensitive [53]. When examining T6SS activity in AB17978 [11], we identified a contact-independent growth inhibition activity. Herein, we follow up on this observation, identify a novel class II microcin (Mcc17978), and provide insights regarding its mechanism of action and regulation.

## Methods

### Bacterial strains and growth conditions

Bacterial strains are listed in Table S1. *Acinetobacter* and *E. coli* strains were grown in Lysogeny broth (LB) at 37 °C with shaking or on LB agar plates. Bacteria were grown in the presence of kanamycin (7.5 µg ml^−1^), gentamicin (10 µg ml^−1^), sulfamethoxazole 30 µg ml^−1^, trimethoprim 6 µg ml^−1^, ampicillin 100 µg ml^−1^, tetracycline 10 µg ml^−1^ or apramycin 25 µg ml^−1^ as needed. Iron limitation was achieved by growing *A. baumannii* and *E. coli* DH5α in Mueller Hinton (MH) medium supplemented with 100 µM, 200 µM, or 300 µM 2,2’-dipyridil (DIP).

### Growth curves

Bacteria were grown overnight in LB medium. Overnight cultures were diluted to an optical density at 600 nm (OD_600_) of 0.05 and aliquots (200 µl) were transferred to 96-well plates in biological duplicates and technical triplicates. The 96-well plates were incubated at 37 °C for 16 h with continuous shaking using a Tecan Spark, Te-Cool, and the Tecan SparkControl Dashboard software. Growth was monitored by OD_600_ and was recorded every ten minutes.

### Molecular cloning

Primers are listed in Table S2. Microcin locus (*ACX60_RS04750-ACX60_RS04770*) was amplified using F_U_*mcc*_500 bp_KO and R_*mcc*_pVRL2 primers and cloned into pVRL2 [54]. Cassettes for *fiu* and *fiuSNP* were amplified from *E. coli* DH5α and *E. coli* DH5α-R1, respectively, using primers F_p*fiu and* R_p*fiu* and cloned into pBAD24. Cassettes for p*mcaBCD* and p*mcaB* were amplified from AB17978 using primers F_*mcaBCD*_pVRL2/R_*mcaBCD*_pVRL2 and F_*mcaB*_pVRL2/R_*mcaB*_pVRL2 respectively. Upstream (1000 bp) and downstream (900 bp) regions of *piuA* from M2 were amplified using primers F_U_piuA_M2_1000 bp/F_R_piuA_M2_1000 bp and F_D_piuA_M2_900 bp/F_D_piuA_M2_900 bp respectively, and cloned into pEX18-Ap. Plasmid pEX18-Ap_UD_piuA_M2 was confirmed by plasmid sequencing. The microcin promoter region containing the predicted fur box was amplified using primers F_*mcc*-luxAB and R_*mcc-*luxAB and cloned into pHK0011. Cloning in pVRL2 was confirmed by PCR and Sanger sequencing using primers F_pVRL2_backbone and R_pVRL2_backbone. Cloning in pBAD24 was confirmed by PCR and Sanger sequencing using primers F_pBAD24_backbone and R_pBAD24_backbone. Plasmids were created using Hi-Fi DNA Assembly mix (New England BioLabs, Ipswich, MA, USA) following manufacturer instructions.

### *E. coli* DH5α chemically competent cells

*E. coli* DH5α competent cells were prepared as previously described [55, 56]. Briefly, bacteria were grown overnight in LB medium. Overnight cultures were refreshed and grown until the density reached ≈ 0.5 OD_600_. Cultures were centrifuged (4300 ***g***, 10 min, 4 °C), resuspended in pre-chilled sterile 100 mM CaCl_2_, and incubated on ice for 30 min. Finally, bacteria were centrifuged (4300 ***g***, 10 min, 4 °C) and resuspended in 100 mM CaCl_2_ in 10 % glycerol.

To transform *E. coli* DH5α competent cells, 50 µl of competent cells were transferred to a microcentrifuge tube with 5 µl plasmid. The cell and plasmid mixture was incubated on ice for 30 min, treated at 42 °C for 45 s and placed on ice for 2 min. Pre-warmed LB medium (1 ml) was added, and the cells were incubated at 37 °C for 1 h with shaking before being plated on LB agar plates with the appropriate antibiotics and incubated at 37 °C overnight.

### *Acinetobacter* electrocompetent cells preparation and transformation

*A. baumannii* and *A. pittii* competent cells were prepared as described [57]. Briefly, bacteria were grown overnight in LB medium. A subculture was grown until reaching an OD_600_ of ≈ 0.5. Cultures were washed twice in cold sterile water and incubated on ice for 10 min. Cells were then washed twice in cold sterile 10 % glycerol and incubated on ice for 10 min. Bacteria were resuspended in 10 % glycerol and stored at −80 °C. Plasmids were introduced by electroporation into the at 2.5 kV cm^−1^, 200 Ω, 25 µF with a GenePulser Cell electroporator (BioRad).

### Construction of *Acinetobacter baumannii* ATCC 17978 mutant strain

The AB17978 ∆*mcc* was constructed as previously described [58]. Briefly, a 1.6 kb kanamycin antibiotic resistance cassette, flanked by FRT flippase recognition sites, was amplified from pKD4 using P1 and P2 primers. Then 500 bp upstream and 600 bp downstream regions of the target locus were amplified from AB17978 genomic DNA, containing flanking regions of the kanamycin cassette. The upstream region was amplified using primers F_U_*mcc*_500 bp_KO and R_U_*mcc*_500 bp_KO primers and the downstream region was amplified using F_D_*mcc*_600 bp_KO and R_D_*mcc*_600 bp_KO primers. A 2.7 kb linear fragment, consisting of the 500 bp upstream fragment, the FRT/kanamycin cassette, and the 600 bp downstream fragment, was obtained by PCR using flanking primers F_U_*mcc*_500 bp_KO and R_D_*mcc*_600 bp_KO. The linear fragment was electroporated into electrocompetent AB17978 cells carrying the plasmid pAT04 expressing RecAB recombinase. Mutants were selected with 7.5 µg ml^−1^ kanamycin, and integration of the linear fragment was confirmed by PCR. To remove the kanamycin resistance cassette, electrocompetent mutants were transformed with pAT03 plasmid, which expresses the FLP recombinase. The mutation was confirmed by Illumina sequencing.

### Construction of *E. coli* MG1655 mutant strain

*E. coli* MG1655 ∆*fiu* deletion mutant was constructed as previously described [59]. Briefly, a 1.6 kb kanamycin antibiotic resistance cassette, flanked by FRT flippase recognition sites, was amplified from pKD4 using primers F_*fiu*_KO and R_*fiu*_KO. The linear fragment was transformed into electrocompetent BW25113 carrying the plasmid pAT04. Mutants were selected on 50 µg ml^−1^ kanamycin, and integration of the linear fragment was confirmed by PCR. BW25113 lysate was prepared using P1 lytic bacteriophage and transduced into MG1655 [60]; the transductants were selected on 50 µg ml^−1^ kanamycin and 100 mM Na-citrate pH 5.5. The resistance cassette was removed by expression of the FLP recombinase from pCP20. The mutant strain was confirmed by PCR using primers F_*fiu*_KO_confirmation and R_*fiu*_KO_confirmation and Illumina sequencing.

### Construction of *Acinetobacter nosocomialis* M2 mutant strain

A clean deletion mutant of *piuA* in M2 was generated as follows: the upstream region was amplified using F_U_piuA_M2_1000 bp and F_R_*piuA*_M2_1000 bp primers and the downstream region was amplified using F_D_*piuA*_M2_900 bp and F_D_*piuA*_M2_900 bp primers. Upstream and downstream fragments were cloned into pEX18-Ap. Biparental mating was carried out using Stellar containing pEX18-Ap_UD_piuA_M2 and M2. Plasmids integrants were selected in chloramphenicol 10 µg ml^−1^ and apramycin 25 µg ml^−1^. Sucrose counterselecion was carried out and candidates were checked by PCR using F_piuA_M2_check and R_piuA_M2_check primers.

### T6SS-independent non-contact proximity spotting assay

Competing bacterial strains were grown overnight in LB to reach stationary phase for 16 h. Bacterial cultures were normalized to OD_600_ 1, washed with LB three times and resuspended in 1 ml of LB. A volume of 10 µl of the microcin producing bacteria was spotted on LB plates. When the spot was dried, a volume of 5 µl was spotted at a distance of 2 mm. The LB plates were incubated at 37 °C for 16 h. Images were taken using the NuGenius gel imaging system (Syngene USA, Frederick, MD, USA). Growth inhibition areas were quantified using the elliptical selection tool and measuring in Fiji 61. We note that the measurement areas we quantified included any spontaneously occurring resistant clones that grew within the inhibition area . MG1655 *∆fiu* containing pBAD24 constructs and DH5α containing pVRL2 constructs were grown overnight and diluted to an OD_600_ of 0.05 in LB medium containing arabinose (0.01 % w/v) and grown for 4 h prior to performing the non-contact proximity spotting assay on LB plates containing arabinose 0.01 % for 16 h. Spotting assays conducted under iron-limiting conditions were performed on MH agar plates containing 0 µM, 100 µM, or 200 µM DIP. Data shown are the mean±SD from three biological triplicates.

### Luciferase assay

AB17978 carrying p*mcc-luxAB* was grown overnight in LB containing 10 µg ml^−1^ tetracycline and diluted to OD_600_ 0.05 in MH, MH +200 µM DIP and MH +200 µM DIP and 100 µM FeCl_3_•6H_2_O for iron replete, iron-limited, and restored iron levels, respectively. In biological triplicates, 90 µl of each culture were incubated in the Tecan with shaking in white, clear bottom 96-well plates at 37 °C for four h, at which time 10 µl of 0.06 % Decanal was injected into the wells. The OD_600_ and Relative Light Unit (RLU) were immediately recorded using a microplate reader Tecan Spark, Te-Cool, and the Tecan SparkControl Dashboard software. Data are presented as the mean value of the relative light units (RLU) normalized to the cell density (OD_600_) with error bars representing one standard deviation.

### Single nucleotide polymorphism (SNP) analysis

Genomic DNA of DH5α-R was isolated using the QIAamp DNA Kit (QIAgen). Illumina sequencing was performed at the Microbial Genome Sequencing Centre (MiGS), and SNPs were detected with Geneious Prime 2021.2.2 (Biomatters, Inc; San Diego, CA, USA) and CLC Genomics Workbench 21 (QIAgen).

### Sanger sequencing

The *fiu* gene was amplified by PCR using *fiu*F1, *fiu*F2 and *fiu*R1 primers from genomic DNA of DH5α-R1. PCR fragments were sequenced by Genewiz (Azenta Life Sciences, South Plainfield, NJ). Data were analysed using Geneious Prime 2021.2.2.

### Statistical analysis

All statistical analyses were carried out with GraphPad Prism 9 software (GraphPad Software, Inc., La Jolla, CA).

### Supporting information

This article contains supporting information: Supplementary Material.

## Results

### AB17978 inhibits *E. coli* DH5α using a non-contact proximity mechanism

**Fig. 1.**
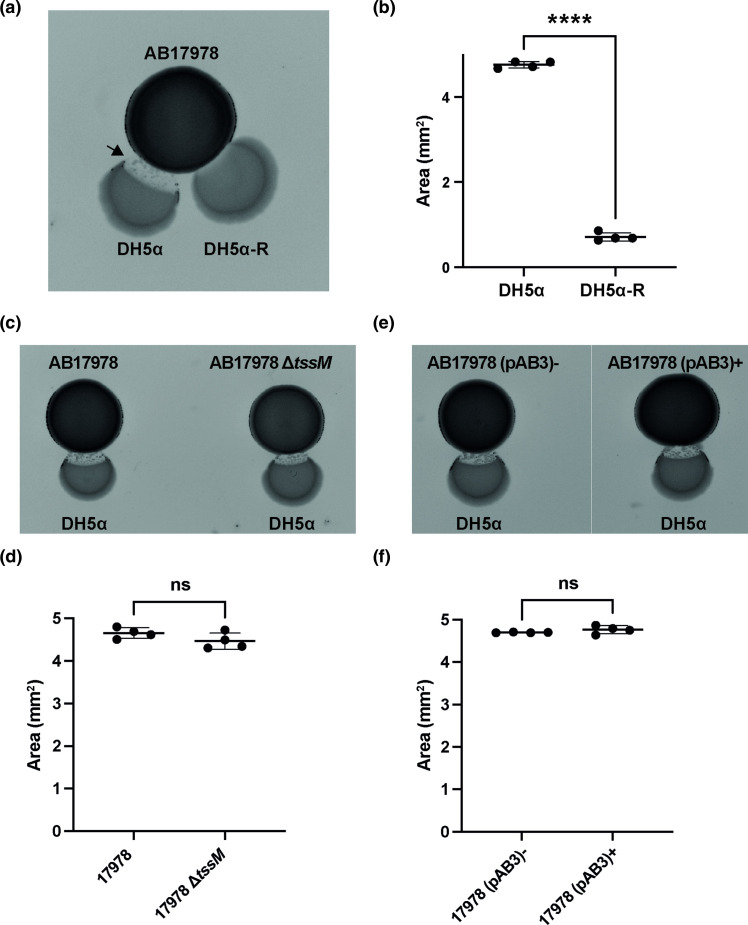
AB17978 inhibits *E. coli* through a non-contact mechanism. (**a**) Proximity spotting assay with AB17978 and *E. coli* DH5α and *E. coli* DH5α-R. Note that DH5α-R was isolated as a spontaneously-occurring resistant colony found within the area of growth inhibition, examples of which are indicated by an arrow. For the proximity spotting assay, the indicated strains were grown in Lysogeny broth (LB) for 16 h to reach stationary phase. Bacterial cells were normalized to an optical density at 600 nm (OD_600_) of 1, washed three times with LB and resuspended in 1 ml of LB. A volume of 10 µl of AB17978 was spotted on a LB plate until the spot dried and then 5 µl of *E. coli* DH5α was spotted at a distance of 2 mm. The plate was incubated at 37 °C for 16 h and images were taken with a gel imaging system. Growth inhibition areas were quantified using the elliptical selection tool and measuring in Fiji. We note that the measurement areas we quantified included any spontaneously occurring resistant clones that grew within the inhibition area. (**b**) Quantification of growth inhibition areas of AB17978 against *E. coli* DH5α and DH5α-R. Data shown are the mean±SD of the growth inhibition areas from two independent experiments in duplicates. ****P<0.0001 (t-test). (**c**) Proximity spotting assay using AB17978 wild-type and AB17978 ∆*tssM* against *E. coli* DH5α. (**d**) Quantification of growth-inhibition areas of *E. coli* DH5α against AB17978 and AB17978 ∆*tssM*. Data are the mean±SD of the growth-inhibition areas from two independent experiments in duplicate. ns=not significant (growth-inhibition areas are significantly different; two-way ANOVA with Tukey Test). (**e**) Proximity spotting assay using AB17978 pAB3− and AB17978 pAB3+ against and *E. coli* DH5α. (**f**) Quantification of growth-inhibition areas of *E. coli* DH5α against AB17978 pAB3− and pAB3+. Data are the mean±SD of the growth-inhibition areas from two independent experiments in duplicate. ns=not significant (growth-inhibition area results are not significantly different; two-way ANOVA with Tukey Test).

We developed a non-contact, proximity spotting assay on solid media to assess growth inhibition between AB17978 and *E. coli* DH5α by a diffusible toxic factor. AB17978 and *E. coli* DH5α were grown in Lysogeny broth (LB) for 16 h to reach stationary phase. Bacterial cells were normalized to an optical density at 600 nm (OD_600_) of 1, washed three times with LB, and resuspended in 1.0 ml of LB. A volume of 10 µl of AB17978 was spotted on an LB plate and allowed to dry. Subsequently, 5 µl of *E. coli* DH5α was spotted at a 2 mm distance and allowed to dry. The plate was incubated at 37 °C for 16 h, and images were taken with a gel imaging system. Growth inhibition areas were quantified using the elliptical selection tool and measuring in Fiji. We note that the measurement areas we quantified included any spontaneously occurring resistant clones that grew within the inhibition area [61] (Fig S1A–C, available in the online version of this article). Results showed an area of growth inhibition in DH5α after 16 h when grown in close proximity to AB17978 (Figs 1a, b and S1A). The antimicrobial activity is T6SS-independent, as AB17978 lacking a functional T6SS (AB17978 *∆tssM*) [11] produces a comparable level of growth inhibition as wild-type AB17978 (Fig. 1c, d). Similar results were observed when using AB17978 with and without pAB3 (Fig. 1e, f), a large conjugative plasmid that represses the T6SS and plays a relevant role in the dissemination of antimicrobial resistance [53, 62]. These results suggest that AB17978 triggers growth inhibition of *E. coli* DH5α through a T6SS-independent, contact-independent mechanism. We next isolated a spontaneously occurring resistant clone of DH5α (DH5α-R) from within the area of growth inhibition during competition with AB17978 (Fig. 1a). Compared to wild-type (WT) DH5α, the resistant clone (called DH5α-R herein) showed a significant reduction in sensitivity to the toxic activity produced by AB17978 (Fig. 1a, b). Whole-genome sequencing combined with variant calling analysis of DH5α-R identified a single nucleotide polymorphism (SNP) in the gene encoding the iron-catecholate transporter Fiu at *T1969G*, which introduces a missense mutation corresponding to amino acid residue substitution Y657D. We confirmed the SNP *T1969G* by Sanger sequencing. As no other mutations were identified in DH5α-R, we hypothesized that the Y657D mutation in the iron-catecholate transporter Fiu protects *E. coli* DH5α from the AB17978 antimicrobial activity.

### A microcin-encoding locus is responsible for the inhibition of *E. coli*

**Fig. 2.**
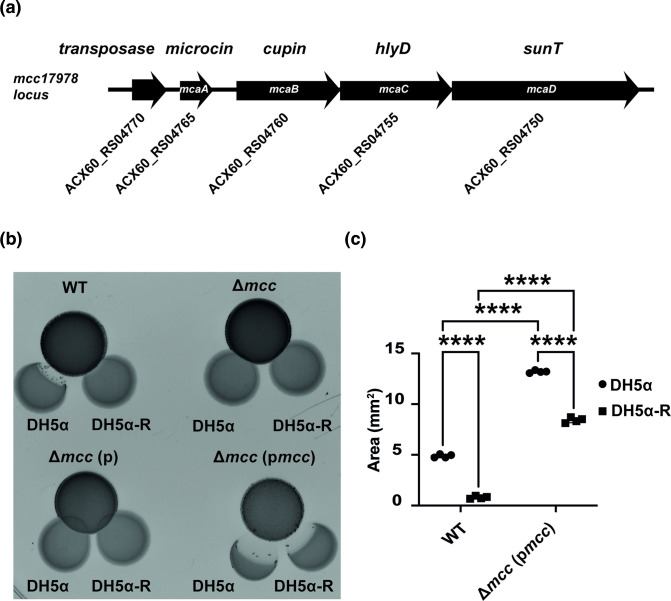
AB17978 encodes a microcin responsible for *E. coli* DH5α inhibition. (**a**) Schematic of the *mcc*-locus encoding the PCAT-type transporter SunT (ACX60_RS04750), HlyD (ACX60_RS04755), Cupin-like protein (ACX60_RS04760), and microcin (ACX60_RS04765). (**b**) Proximity spotting assay using AB17978 WT, ∆*mcc*, ∆*mcc* (**p**), and ∆*mcc* (p*mcc*) against *E. coli* DH5α WT and resistant DH5α-R. (**c**) Quantification of growth inhibition areas of AB17978 WT and ∆*mcc* (p*mcc*) against *E. coli* DH5α and DH5α-R. Data shown as the mean±SD of the growth inhibition areas from two independent experiments in duplicate. ****P<0.0001 (two-way ANOVA with Tukey’s test for multiple comparisons). ∆*mcc* (**p**), ∆*mcc* strain harbouring empty vector pVRL2; ∆*mcc* (p*mcc*), ∆*mcc* strain harbouring pVRL2 carrying the microcin locus.

The involvement of Fiu led us to hypothesize that the antagonistic activity may be due to a microcin, as Fiu-type iron transporters are targeted by several microcins, including the class IIb microcins MccH47, MccM, and MccE492 [13, 15, 27] and the class IIa microcin MccN [19]. As class II microcins are secreted through PCATs such as SunT [63], we performed a blast nucleotide (BLASTn) search of the AB17978 genome for SunT and identified a locus spanning several genes (*ACX60_RS04750 to ACX60_RS04770*) encoding a putative SunT homolog (*ACX60_RS04750*), a hemolysin secretion protein D (HlyD) (*ACX60_RS04755*), a cupin-like gene (*ACX60_RS04760*), and a putative microcin (*ACX60_RS04765*) (Fig. 2a). The predicted microcin carries a leader peptide ending with a GA motif at residues 15–16 (Fig. S2A), similar to the bactericidal class IIb microcins MccH47 and MccE492 and class IIa microcins MccV, MccL and MccN [14]. This locus is also present in other *A. baumannii* strains as part of a Tn6171-derived mobile element [51] (Fig. S3).

We constructed a strain harbouring a deletion of the entire locus, which we refer to here as ∆*mcc*. The ∆*mcc* strain did not display antimicrobial activity against DH5α in our proximity plating assay (Figs 2b, c and S2B). Importantly, the lack of the microcin locus did not affect bacterial growth (Fig. S2C). Complementation of the ∆*mcc* strain with a plasmid containing the full *mcc* locus (p*mcc*) restored growth inhibition against both DH5α and DH5α-R (Figs 2b, c and S2B). We suspect the enhanced activity is a result of overexpression from the multi-copy plasmid backbone (pVRL2) used for complementation (Figs S2B, S2D). Taken together, we conclude that the putative microcin locus identified here encodes the genes necessary for growth inhibition of *E. coli* DH5α. We further purpose to name these genes *mcaABCD*, for microcin locus in *Acinetobacter*, with *mcaA* encoding Mcc17978, *mcaB* the cupin-like gene, *mcaC* the *hlyD-*homolog and *mcaD* the SunT homolog.

### The *mcc* locus is sufficient to confer contact-independent inhibition

**Fig. 3.**
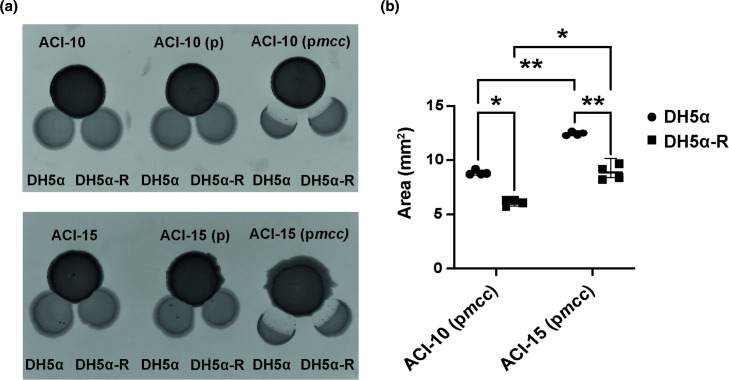
Expression of the *mcc* locus in *A. baumannii* ACI15 and *A. pittii* ACI10 enables contact-independent inhibition of *E. coli* DH5α. (**a**) Proximity spotting assay using derivative strains of *A. pittii* clinical isolate ACI10 (top) and *A. baumannii* clinical isolate ACI15 (bottom) against *E. coli* DH5α and DH5α-R. (**b**) Quantification of growth inhibition areas of *E. coli* DH5⍺ and DH5⍺-R against ACI10 (p*mcc*) and ACI15 (p*mcc*). Data are the mean±SD of the growth inhibition areas from two independent experiments in duplicate. ACI10 (p*mcc*):DH5α vs ACI15 (p*mcc*):DH5α **p = (0.0097), ACI15 (p*mcc*):DH5α vs ACI15 (p*mcc*):DH5α-R **p = (0.01), ACI10 (p*mcc*):DH5α vs ACI10 (p*mcc*):DH5α-R *p = (0.0205), ACI10 (p*mcc*):DH5α-R vs ACI15 (p*mcc*):DH5α-R *p = (0.0197) (two-way ANOVA with Tukey’s test for multiple comparisons).

To further demonstrate that the *mcc* locus is responsible for the antagonistic activity produced by AB17978, we tested whether the *mcc* locus alone was sufficient to lead to growth inhibition of DH5α. We introduced the *mcc* plasmid (p*mcc*) into two *Acinetobacter* clinical isolates that lack the *mcc* locus. Neither *A. baumannii* ACI15 nor *Acinetobacter pittii* ACI10 exhibit antagonistic activity towards DH5α in our proximity spotting assay (Fig. 3a). On the contrary, when we introduced plasmid p*mcc* into these strains, both demonstrated antimicrobial activity against *E. coli* DH5α (Fig. 3a, b), with ACI10 (p*mcc*) having less antimicrobial activity against *E. coli* compared to ACI15 (p*mcc*) (Figs 3a, b and S4). These results demonstrate that the *mcc* locus identified herein encodes the necessary components to confer non-contact dependent growth inhibition.

### Wild-type Fiu, but not Fiu Y657D, restores Mcc17978 sensitivity

**Fig. 4.**
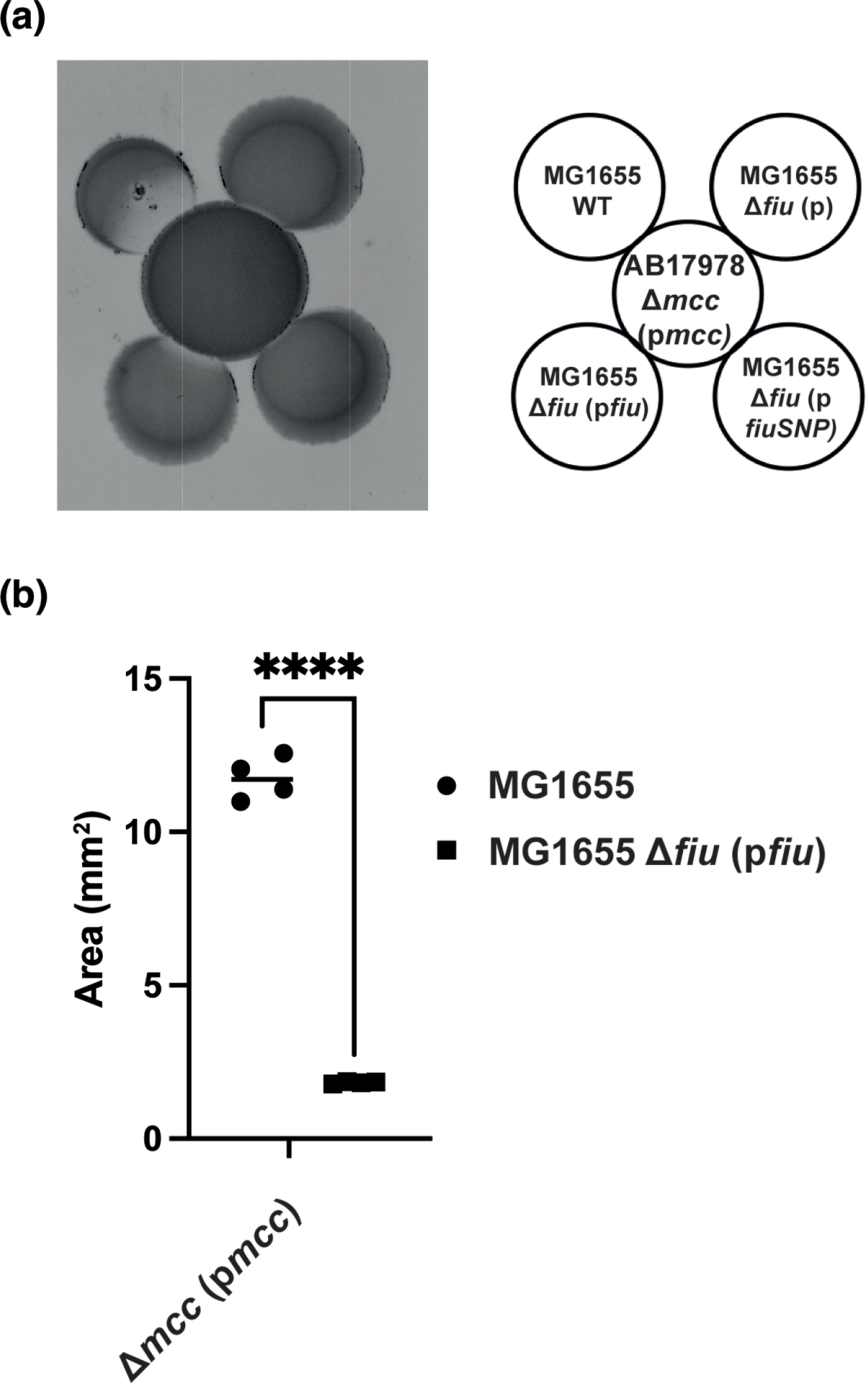
The *fiu* allele *fiuSNP* protects MG1655 from Mcc17878 activity. (**a**) Proximity spotting assay using ∆*mcc* (p*mcc*) against MG1655 WT, ∆*fiu* (**p**), ∆*fiu* (p*fiu*), and ∆*fiu* (p*fiuSNP*). Expression of Fiu and FiuSNP was induced with 0.01 % arabinose. (**b**) Quantification of growth inhibition areas of AB17978 WT and ∆*mcc* (p*mcc*) against MG1655 and MG1655 ∆*fiu* (p*fiu*). Data shown are the mean±SD of the growth inhibition areas from two independent experiments in duplicate. ****P<0.0001 (two-way ANOVA with Tukey’s test for multiple comparisons), ∆*fiu* (**p**), MG1655 ∆*fiu* strain harbouring empty pBAD24 vector; ∆*fiu* (p*fiu*), MG1655 ∆*fiu* strain harbouring wild-type *fiu* in pBAD24; ∆*fiu* (p*fiuSNP*), MG1655 ∆*fiu* strain harbouring the *fiu-T1696G* allele in pBAD24.

As discussed above, the *microcin-*resistant DH5α-R isolate harboured a single point mutation in the *fiu* coding sequence; here we refer to the *fiu* gene variant as *fiuSNP* and the protein variant as FiuSNP. To further explore the possibility that Fiu is the receptor for Mcc17978, we performed our proximity spotting assay with ∆*mcc* (p*mcc*), which exhibits a high growth inhibition phenotype, against a different *E. coli* strain, strain MG1655. When cultured in proximity to ∆*mcc* (p*mcc*), the growth of wild-type MG1655 was inhibited (Fig. 4a). MG1655 ∆*fiu* was fully protected from the antimicrobial activity of ∆*mcc* (p*mcc*). Furthermore, while MG1655 *∆fiu* (*pfiuSNP*) was fully protected against ∆*mcc* (p*mcc*)*,* MG1655 ∆*fiu* (*pfiu*) was sensitive to ∆*mcc* (p*mcc*) (Figs 4a, b and S5A). Importantly, neither MG1655 cells lacking *fiu* (MG1655 ∆*fiu*) nor ∆*fiu* cells harbouring plasmids with the wild-type *fiu* or *fiuSNP* had a visible growth defect (Fig. S5B). We thus conclude that FiuSNP confers protection to the antimicrobial activity elicited by Mcc17978.

### Iron regulates the microcin system in AB17978

**Fig. 5.**
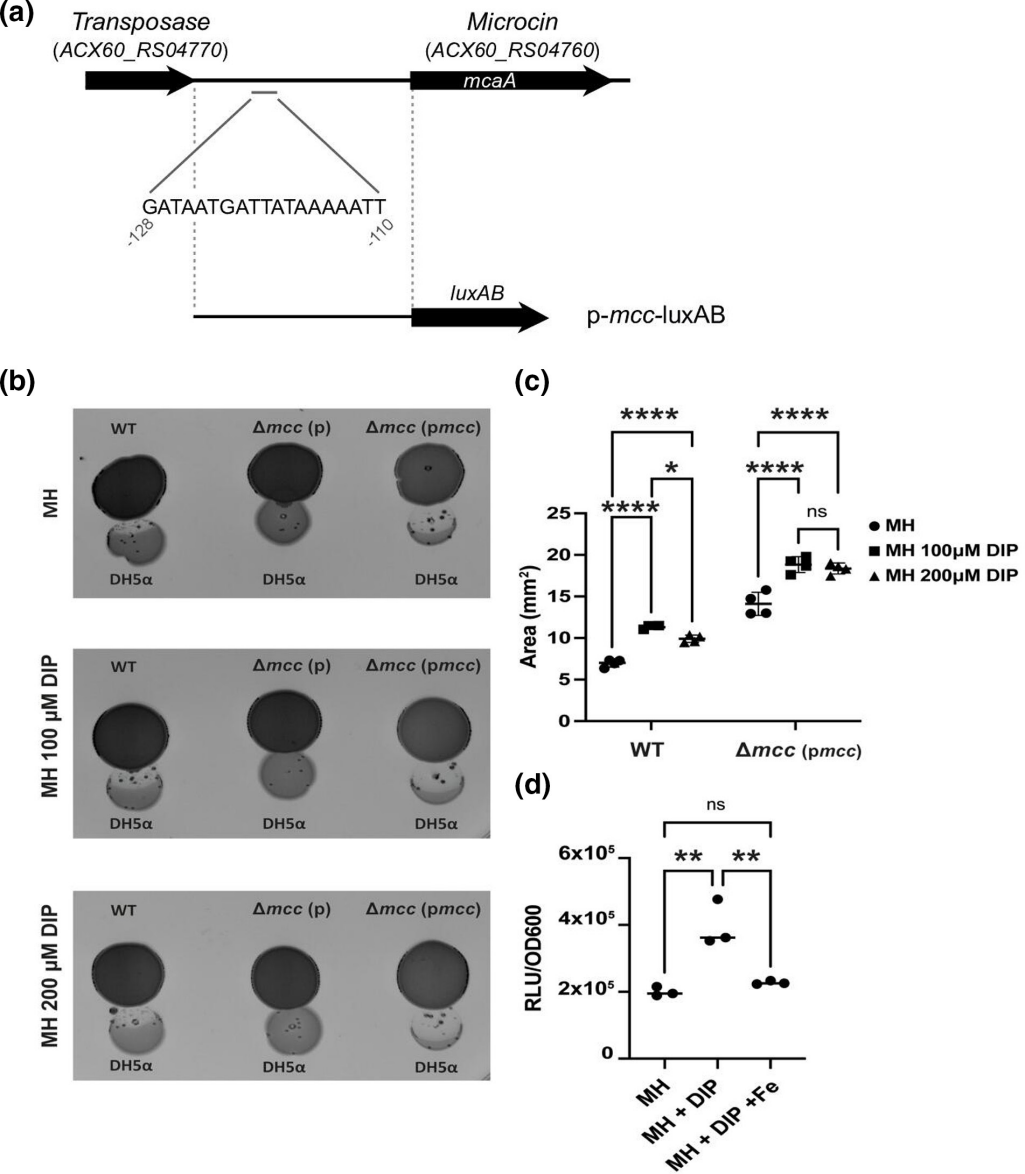
Expression of the *mcc* locus responds to iron availability. (**a**) Schematic of the microcin promoter region showing the sequence and location of the putative Fur box (grey bar; at nucleotide position −128 to −110 relative to the start codon of the microcin gene). The *mcc* promoter region included in plasmid p*mcc*-luxAB is indicated with dashed grey lines. (**b**) Proximity spotting assay with AB17978 WT, ∆*mcc* (**p**) and ∆*mcc* (p*mcc*) against DH5α in MH media (top panel), MH containing 100 µM DIP (middle panel) and MH containing 200 µM DIP (bottom panel). (**c**) Quantification of growth inhibition areas for AB17978 WT and ∆*mcc* (p*mcc*) against *E. coli* DH5α and DH5α-R. Data shown are the mean±SD of the growth inhibition areas from two independent experiments in duplicate. ****P<0.0001, *p = (0.0101), ns=not significant (two-way ANOVA with Tukey’s test for multiple comparisons). (**d**) Luciferase AB (*luxAB*) reporter assay monitoring *mcc* promoter region, including the Fur box, in MH media, MH containing 200 µM DIP and MH containing 200 µM DIP and 100 µM iron. Data shown are the mean±SD of the Relative Light Unit (RLU) normalized to OD_600_ from three biological triplicates. MH vs MH +DIP **p = (0.0025), MH +DIP vs MH +DIP+ Fe **p = (0.005), ns=not significant (One-way ANOVA with Tukey’s test for multiple comparisons).

Microcin expression is often linked to iron availability and is regulated by Fur in other systems [39, 40]. Thus, we searched for a putative Fur binding box and identified GATAATGATTATAAAAATT [41] upstream of the start codon for the microcin gene at position −128 to −110 relative to the start codon of *mcaA* (Fig. 5a). We assessed the growth inhibition activity of AB17978 WT and ∆*mcc* (p*mcc*) under various iron conditions. As shown in Fig. 5, panels B and C, we observed greater growth inhibition activity when we performed the experiments in the presence of increasing concentrations of the iron chelator 2,2’-dipyridil (DIP). Importantly, the ∆*mcc* strain harbouring the empty vector pVRL2, ∆*mcc* (p), did not show antimicrobial activity in any of the tested conditions (Figs 5b, c and S6).

We next assessed the activity of the *mcc* promoter using a luciferase-based reporter assay. The activity of the microcin promoter reporter increased significantly upon iron depletion (i.e. Mueller Hinton agar [MH] with 200 µM DIP) compared to cells grown in either MH alone or in MH containing both the chelator and exogenous free iron (Fig. 5d). The growth of AB17978 and DH5α was unaffected under the iron-limited conditions tested here (Fig. S7). The growth of ∆*mcc* (p*mcc*) was, however, affected at increasing concentrations of chelator. We speculate that this results from excessive expression of Mcc17978 from the plasmid, overwhelming the native Mcc17978 immunity system (Figs S2D and S7D). Taken together, these data show that the microcin cluster is upregulated under iron-limiting conditions and is potentially under control of the Fur transcription regulator.

### The microcin locus confers protection against Mcc17978 antimicrobial activity

**Fig. 6.**
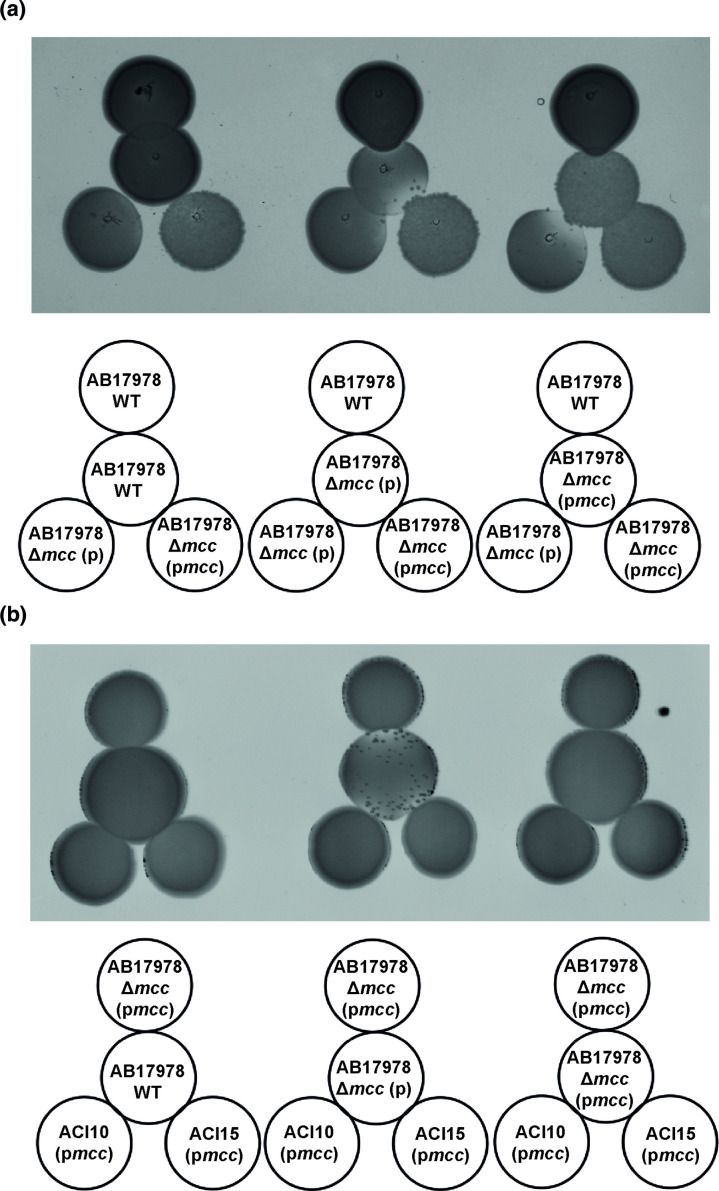
The microcin locus confers protection against Mcc17978 antimicrobial activity. (**a**) Proximity spotting assay with AB17978 WT, ∆*mcc* (**p**), and ∆*mcc* (p*mcc*) against AB17978 WT, ∆*mcc* (**p**), and ∆*mcc* (p*mcc*). (**b**) Proximity spotting assay with AB17978 WT, ∆*mcc* (**p**), and ∆*mcc* (p*mcc*) against ∆*mcc* (p*mcc),* ACI10 (p*mcc*), and ACI15 (p*mcc*).

Microcin production is toxic to the producing bacterial cells and to kin cells if self-immunity strategies are not available [13, 15, 27, 38, 63, 64]. In agreement with our previous findings, in a proximity spotting assay, ∆*mcc* (p*mcc*) inhibited the ∆*mcc* strain; possibly due to the combination of Mcc17978 overexpression in the complemented strain background and the absence of an immunity system in the null background. Additionally, AB17978 WT showed antimicrobial activity against the ∆*mcc* strain under conditions where *mcaA* expression is increased (i.e. low iron conditions, Fig. S6). On the contrary, AB17978 WT and ∆*mcc* (p*mcc*) were immune to the microcin activity of ∆*mcc* (p*mcc*) (Fig. 6a). We further tested if trans-complementation of the microcin locus in ACI10 and ACI15 resulted in antimicrobial activity against the AB17978 ∆*mcc* mutant in a proximity spotting assay and found that, indeed both ACI10 (p*mcc*) and ACI15 (p*mcc*) could inhibit the growth of the *∆mcc* strain (Fig. 6b, middle). Moreover, ACI10 (p*mcc*) is protected against AB17978 microcin activity (Fig. 6b, right; Fig. S8). These data support the hypothesis that the Mcc17978 locus provides immunity functions to protect against Mcc17978 activity, preventing self-intoxication in strains harbouring the *mcc* locus.

**Fig. 7.**
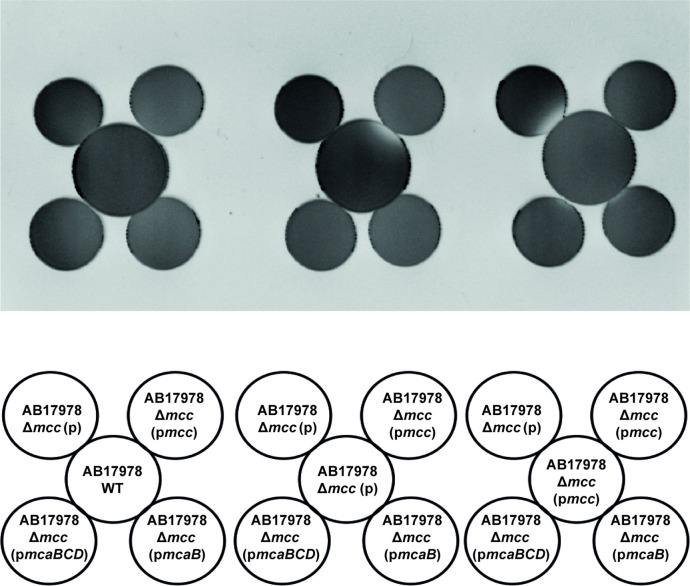
The cupin-like protein encoded by *mcaB* is sufficient to prevent Mcc17978 toxicity. Proximity spotting assay with AB17978 WT, ∆*mcc* (**p**), and ∆*mcc* (pmcc) against ∆*mcc* (**p**), ∆*mcc* (p*mcc*), ∆mcc (p*mcaBCD*) and ∆mcc (p*mcaB*).

To further investigate the immunity system for Mcc17978, we next asked if any of the three downstream genes of the *mcc* locus, which encode for a cupin-like protein (*mcaB*) and two putative transport proteins (*mcaC* and *mcaD*), play a role in resisting Mcc17978 activity. As shown in Fig. 7, when harboured on a plasmid, the three downstream genes (p*mcaBCD*) or *mcaB* alone (p*mcaB*) protected the ∆*mcc* mutant from the antibacterial activity produced by Δ*mcc* (p*mcc*). These results suggest that the cupin-like protein encoded by *mcaB* is sufficient to prevent Mcc17978 toxicity and may thus be a key component of the immunity system to prevent self-intoxication from Mcc17978.

### AB17978 microcin antagonizes other *Acinetobacter* isolates

**Fig. 8.**
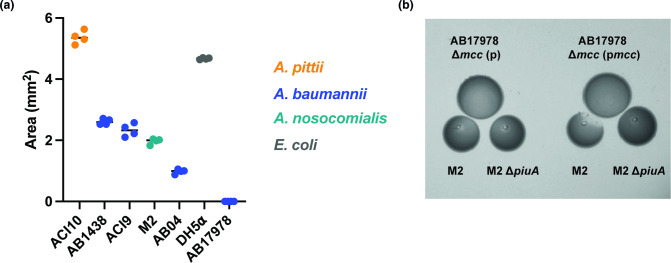
AB17978 inhibits growth of other *Acinetobacter* clinical isolates. (**a**) Quantification of growth inhibition areas of *A. pitti* (orange circles) strain ACI10, *A. baumannii* (blue circles) strains AB1438, ACI9, and AB04, and *A. nosocomialis* (green circles) strain M2, in a proximity spotting assay against AB17978. Inhibition areas of DH5α (grey, circles) and AB17978 are included as controls. Data are the mean±SD of the growth inhibition areas from two independent experiments performed in duplicate. (**b**) Proximity spotting assay with ∆*mcc* (**p**), and ∆*mcc* (pmcc) against M2 and M2 ∆*piuA*.

We tested if the Mcc17978 system provides a competitive advantage for AB17978 against other *Acinetobacter* isolates. Using the proximity spotting assay, we demonstrated that Mcc17978 exhibits growth inhibition activity against other *A. baumannii* strains and *Acinetobacter* species, including *A. pittii* strain ACI10, *A. baumannii* strains AB1438, ACI9, AB04, and *A. nosocomialis* strain M2 (Figs 8A and S9). *A. baumannii* genomes encode for a putative iron receptor called *piuA* (*ACX60_RS15725 i*n AB17978) [65], which belongs to the same clade as *fiu*. We constructed a strain harbouring a deletion of *piuA* (locus tag *FDQ49_RS07115*) in M2, and determined that M2 ∆*piuA* was fully protected from the antimicrobial activity of ∆*mcc* (p*mcc*) (Fig. 8b). We also found several *Acinetobacter* resistant strains, including, ACI1, ACI2, ACI3, ACI6, ACI7, ACI14, ACI16, and 1225 AbCAN2, a finding that suggests that Mcc17978 may have a limited spectrum of activity against other *Acinetobacter* isolates or that these strains employ mechanisms to resist intoxication with Mcc17978 (Fig. S10). Taken together, our data indicate that AB17978 can inhibit the growth of other *Acinetobacter* strains, suggesting that the Mcc17978 locus may provide a competitive advantage during infection.

## Discussion

The nosocomial pathogen *A. baumannii* has evolved contact-dependent and contact-independent strategies to compete with neighbouring bacteria. In this study, we uncovered a T6SS-independent competition activity employed by AB17978 to compete with other *Acinetobacter* isolates and multiple strains of *E. coli *. This inhibitory activity is due to a previously uncharacterized class II microcin which we term Mcc17978. We isolated a resistant clone of *E. coli* DH5α and found that this strain harboured a single nucleotide change (*T1969G*), leading to a single residue change in the iron-catecholate receptor Fiu (Fiu-Y657D) (Fig. 1a). Moreover, when we complemented a *fiu* null strain with the *fiuSNP* allele, the resulting strain was immune to Mcc17978 activity (Fig. 4), suggesting that Fiu is likely a receptor for Mcc17978. Our finding of Fiu as a potential receptor for Mcc17978 is consistent with previous findings of Fiu being the receptor for several class IIb microcins, including microcins MccH47, MccM, and MccE492 [13, 15, 27, 66] and one class IIa microcin, microcin MccN [19]. Two mechanisms might explain the resistance of FiuSNP to the growth inhibition activity produced by AB17978. A change of tyrosine (Y) to aspartate (D) might alter electrostatic interactions between Fiu and Mcc17978; alternatively, the Y657D SNP could lock Fiu in the closed state [20]. Both possibilities would likely limit the transport of Mcc17978 into the target cell, reducing its antimicrobial capacity. We identified a Fiu homolog in *A. nosocomialis* strain M2 [65], PiuA. Lack of *piuA* protected M2 from the growth inhibition activity from ∆*mcc* (p*mcc*) (Fig. 8b).

We further determined that the genes responsible for the inhibitory activity are encoded by the *mcaABCD* locus (*ACX60_RS04750-ACX60_RS04770*) as cells lacking this locus (herein termed ∆*mcc*) no longer confer antimicrobial activity (Fig. 2b, c). Notably, when harbouring a plasmid encoding the *mcc* locus, both the ∆*mcc* mutant strain and *Acinetobacter* strains that otherwise lack the microcin locus (* A. baumannii* ACI15 and *A. pittii* ACI10), exhibit a robust antibacterial phenotype against both *E. coli* (Figs 2–4) and other *Acinetobacter* species (Fig. 6b). The microcin system in AB17978 is located in Tn6552, a Tn7-like derivative of Tn6171 that was first described in *A. baumannii* D36 [51, 67]. Additional Tn6171 derivatives were found in other *A. baumannii* strains, including *A. baumannii* ABPK1, 5457, VB2486, and ABCTX13, suggesting that while the locus is narrowly spread within the *Acinetobacter* genus, it is present in diverse lineages from each of the two dominant clonal groups (global clones 1 and 2) responsible for causing difficult to treat *A. baumannii* infections across the world [51].

Previous studies report that microcin production is relevant in the host environment [68, 69] where iron is often limiting. While microcins are not restricted to pathogenic strains, it has been proposed that pathogenic bacteria employ microcins to compete with other bacteria and promote their survival under the low iron conditions found in the host environment [41, 51, 70]. For example, microcin MccE492 is regulated by iron availability through the iron-responsive Fur regulator [39]. In *A. baumannii *, Fur positively regulates siderophore clusters under iron-limiting conditions [41], and microarray data revealed that the *cupin* and *hlyD* genes located within the *mcc* locus were upregulated under low iron availability [41]. Interestingly, in addition to the microcin cluster, Tn6552 also encodes a siderophore system [48]. In line with these previous findings, we provide experimental data showing that Mcc17978 production is enhanced during low iron conditions (Fig. 5).

Class II microcin producer cells express immunity proteins that partially or fully protect themselves from antimicrobial activity; however, these self-immunity mechanisms remain largely uncharacterized [13, 15]. It has been speculated that the variable region at the N-terminus of the mature microcin may be involved in specific immunity through interaction with immunity proteins to counteract the antimicrobial activity [15]. For example, class IIb microcin MccH47 is neutralized by MchI, encoded in the same locus as MccH47 and anchored in the cytoplasmic membrane [38]. Another class IIB microcin, MccE492, is prevented from interacting with its target mannose permease by immunity protein MceB, which also localizes to the producer cell’s inner membrane [34, 35]. According to our data, strain AB17978 encodes an immunity system to protect the producing cell against self- and kin-competition. Our data suggest that the cupin-like protein encoded by *mcaB* functions as an immunity protein, as the ∆*mcc* strain harbouring *mcaB-*containing plasmids are protected from Mcc17978 activity (Fig. 7).

Moreover, *A. pittii* expressing the microcin locus from an exogenous plasmid is protected from the inhibitory activity of AB17978 (Fig. S8 top panel). It is also possible, however, that strain AB17978 harbours a basal intrinsic resistance to Mcc17978, as ∆*mcc* is not affected by the microcin levels produced by AB17978 WT (Fig. 6a, left). As mentioned above, we showed that PiuA is the receptor for Mcc17978 in M2. Mcc17978-sensitive strains (* A. pittii *, *A. baumannii* AB04) also encode a PiuA homolog. Interestingly, Mcc17978-resistant strain 1225 AbCAN2 lacks the *mcc* locus but does encode a PiuA homolog (QHB89173.1), suggesting that this particular strain may have evolved an alternative strategy to counteract intra-* Acinetobacter* competition. Whole genome sequencing for strains ACI1, ACI2, ACI3, ACI6, ACI7, ACI14, and ACI16 has not been performed, so we are unable to determine if these strains encode for the *mcc* locus or if they encode a *piuA* homolog (Fig. S10).

Microcins are narrow-spectrum antimicrobials, mostly produced by *Enterobacteria*, mainly *E. coli* [68]. Here, we studied for the first time a microcin produced by AB17978 (order of *Pseudomonadales *) that can trigger competition with closely related *Acinetobacter* strains and non-closely related *E. coli* strains.

**Fig. 9.**
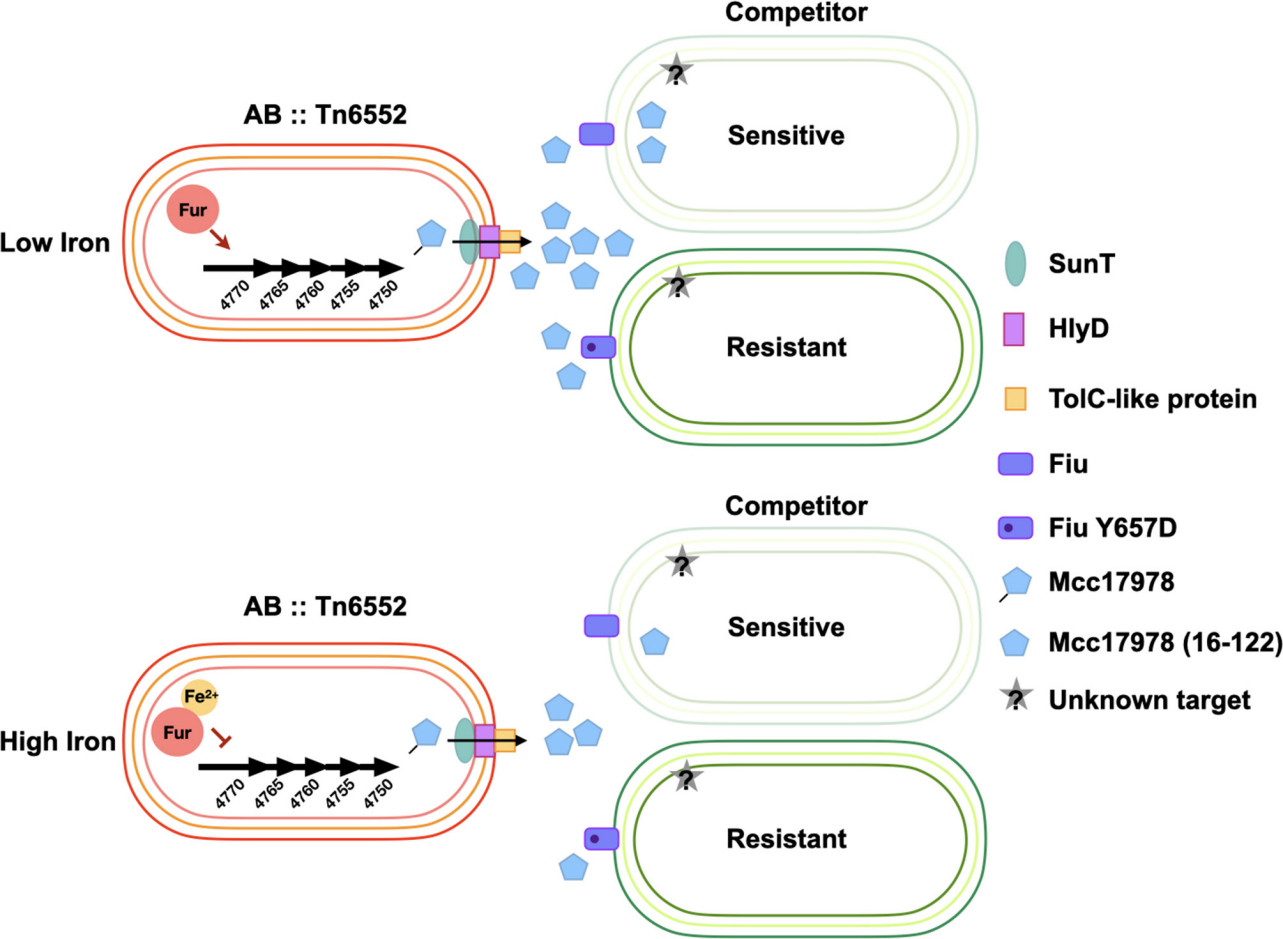
Model of T6SS-independent inhibition mediated by AB17978 and Mcc17978. At low iron levels, Fe^2+^ is not available to form repressor complexes with Fur (Fur•Fe^2+^) and the Fur box located upstream the microcin gene, leading to de-repression of the *mcc* operon. Following secretion, Mcc17978 binds to and enters target cells through Fiu, triggering antimicrobial activity (top panel). At high iron levels, Fur•Fe^2+^ complexes repress expression of the microcin locus, and antimicrobial activity against target cells is downregulated (bottom panel).

Based on our results, we propose the model shown in Fig. 9 for the AB17978 microcin-mediated inhibition of *E. coli *, which depends on iron availability. When iron concentration is low, Fur does not repress the microcin locus. Mcc17978 is expressed, exported, and ultimately interacts with the iron-catecholate transporter Fiu on target *E. coli* cells and inhibits their growth. We further hypothesize that the PCAT transporter SunT recognizes and translocates Mcc17978 into the periplasmic space, where the HlyD periplasmic adaptor protein and the outer membrane protein TolC secretes the microcin into the extracellular space. Mcc17978 kills susceptible competitor strains, possibly by interacting with transporters in the outer membrane, such as Fiu in *E. coli* or PiuA in *Acinetobacter* strains. Our model thus proposes that the uptake of Mcc17978 ultimately triggers growth inhibition in the intoxicated cell. In contrast, at higher iron levels, Fe^2+^ binds to Fur, which represses transcription of the microcin locus and decreases contact-independent proximity inhibition against competing bacteria.

This work constitutes the first demonstration of the presence and production of a microcin in *Acinetobacter *. It is tempting to speculate that additional microcins will be discovered in this genus. Additional work is needed to determine whether Mcc17978 belongs to an existing subgroup of class II microcins (i.e. class IIa or IIb) or if it belongs to a novel subgroup of class II mircocins. Moreover, further experimentation will be needed to more fully address the molecular basis of self-immunity to Mcc17978. Finally, it remains to be seen if there are specific post-translational modifications that impact the production, export, or entry efficiency through target receptors; all of which factors may alter interbacterial competition.

## Supplementary Data

Supplementary material 1
