# Supplementary material for: Acinetobacter baumannii ATCC 17978 encodes a microcin system with antimicrobial properties for contact-independent competition
Source: Microbiology (Reading). 2023 Jun 7;169(6):001346. doi: 10.1099/mic.0.001346 (PMC10333792; doi:10.1099/mic.0.001346)
Supplement: Supplementary material 1 [file mic-169-1346-s001.pdf]

Acinetobacter baumannii ATCC 17978 encodes a microcin system with antimicrobial properties for contact-independent competition

Fabiana Bisaro[1,3], Howard Shuman[2,✝], Mario F. Feldman[3], Michael Gebhardt[4*] Stefan Pukatzki[1*].

Contents:

Supplementary Figures S1-S10

Supplementary Table S1: Strains and Plasmids

Supplementary Table S2: Primers

Supplementary Material and Methods

Supplementary references

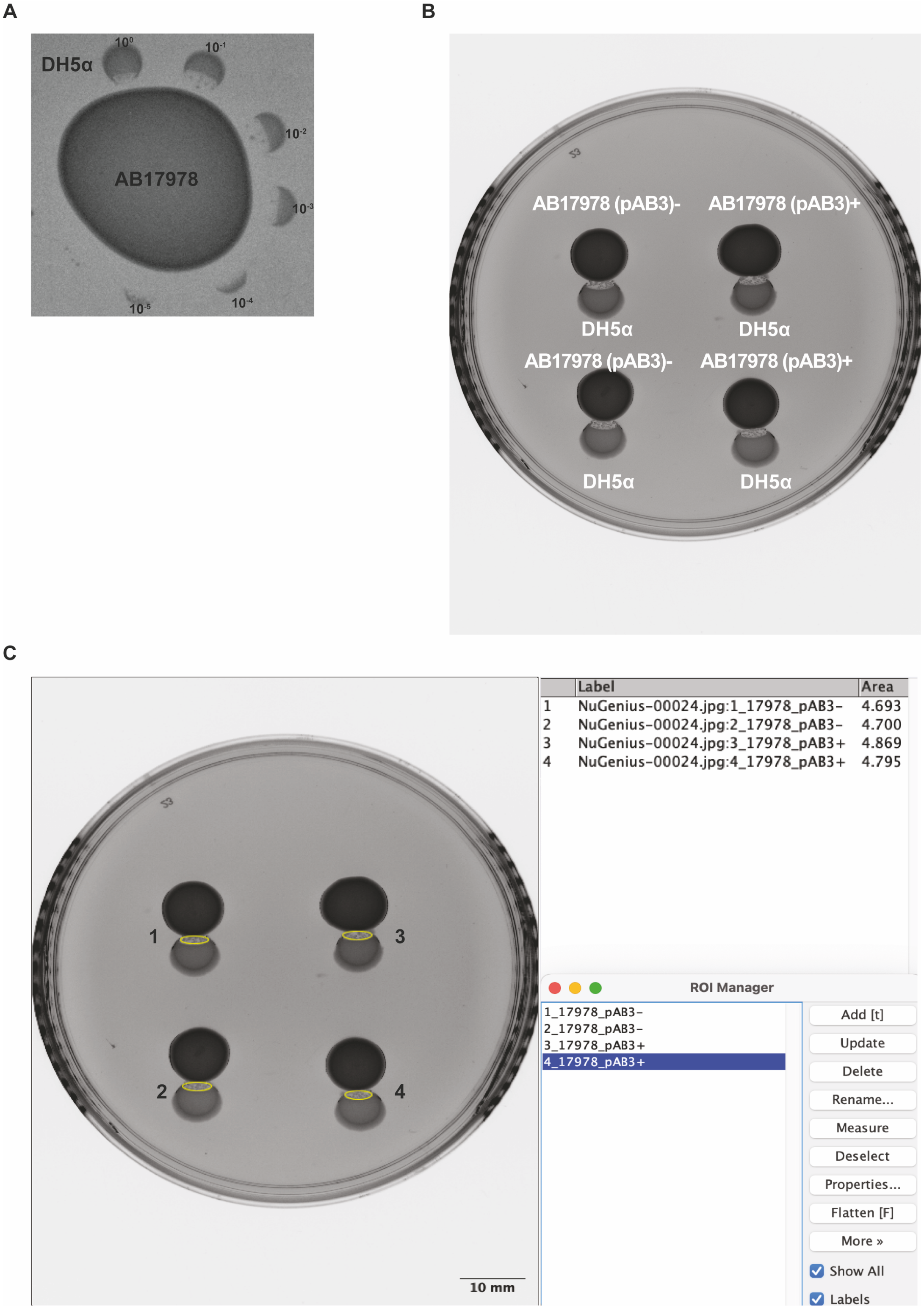

FIG S1. AB17978 inhibits *E. coli* DH5α in a T6SS-independent manner.

(A) Spotting assay with no contact between spots using AB17978 wild type against *E. coli* DH5α dilutions of 10^0, 10^{-1}, 10^{-2}, 10^{-3}, 10^{-4}, 10^{-5}.

(B) Image obtained with NuGenius gel imaging system (Syngene USA, Frederick, MD, USA), used for quantification of growth inhibition areas in Fiji.

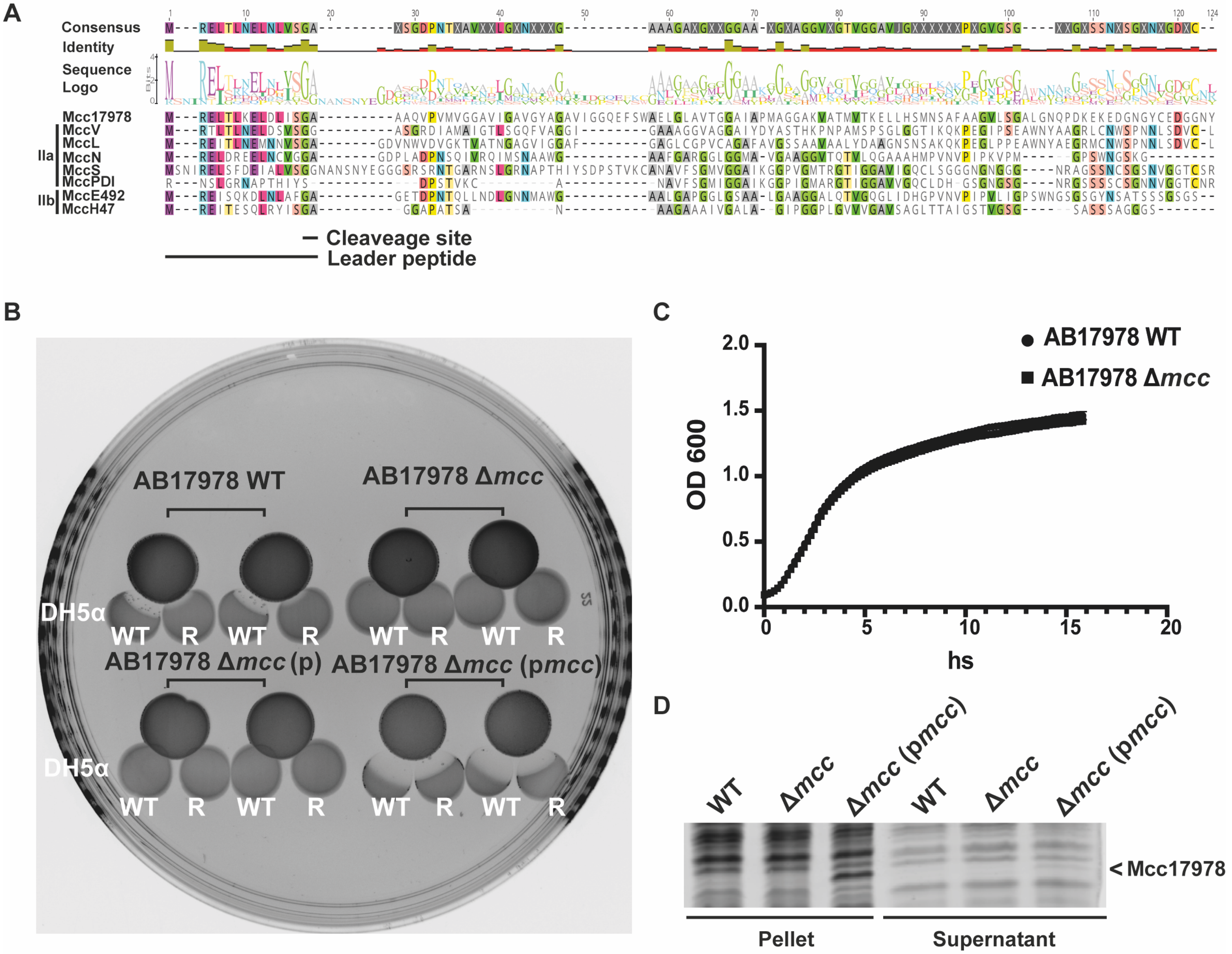

(C) Quantification of growth inhibition areas of Fig S1B, using the elliptical selection tool and measuring in Fiji. The measurement areas we quantified included any spontaneously occurring resistant clones grew within the inhibition area. Scale bar is shown.

FIG S2 AB17978 contains a locus that encodes the microcin responsible for antimicrobial activity against *E. coli* DH5α.

(A) Protein alignment of Mcc17978 against class IIa and class IIb microcins. Cleavage site Glycine$_{15}$-Alanine$_{16}$ (GA) is shown. The leader peptide of Mcc17978 was predicted by SignalP.

(B) Biological and technical duplicates of proximity spotting assay using AB17978 and derivative strains, Δ*mcc,* Δ*mcc* (p), and Δ*mcc* (p*mcc*), against *E. coli* DH5α and DH5α-R used for quantification in Figure 2C.

(C) Growth curves of AB17978 WT and Δmcc.

(D) Coomassie blue staining of total protein and filtered supernatant samples from AB17978 WT, Δ*mcc*, and Δ*mcc* (p*mcc*). Mcc17978 is indicated by an arrow.

A

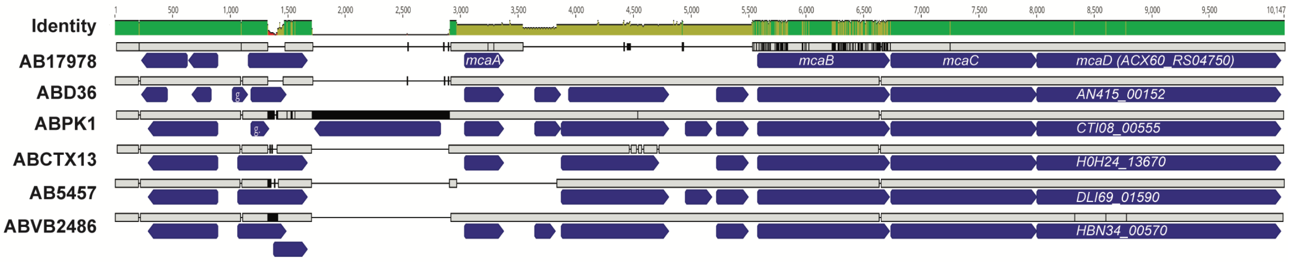

B

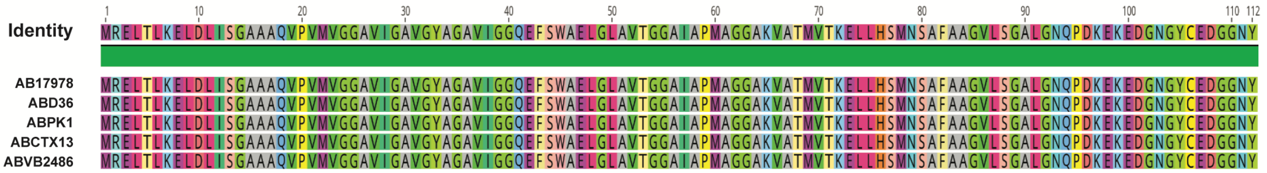

FIG S3 Microcin locus in *Acinetobacter baumannii* strains.
(A) Nucleotide alignment of microcin locus in AB17978, D36, ABPK1, ABCTX13, AB5457, and ABVB2486. It is indicated in AB17978 *mcaA* encoding Mcc17978, *mcaB* the cupin-like gene, *mcaC* the hlyD-homolog and the SunT homolog *mcaD*
(B) Microcin protein sequence alignment from AB17978, D36, ABPK1, ABCTX13, and ABVB2486, (top panel). Amino acid sequence of microcin proteins is 100% identical. AB5457 lacks the microcin gene.

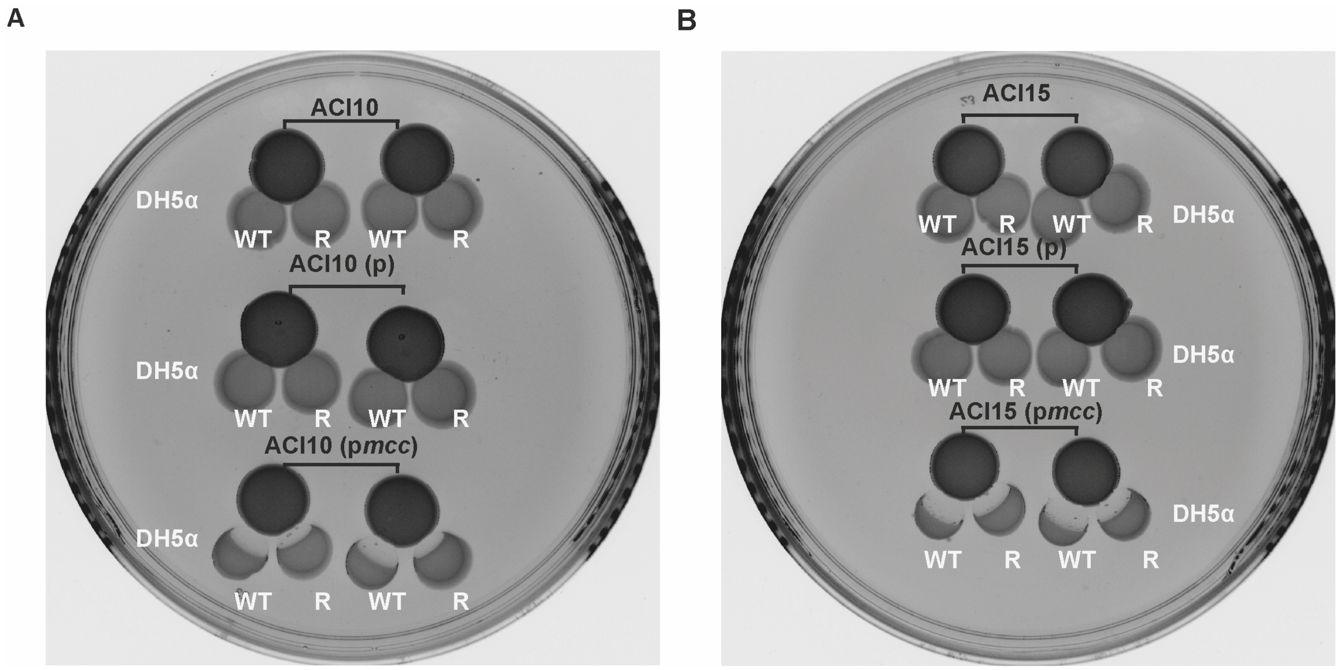

FIG S4 Proximity spotting assay using derivative strains of ACI10 and ACI15 against *E. coli* DH5α and *E. coli* DH5α-R

(A) Proximity spotting assay of ACI10*,* ACI10 harboring the empty vector ACI10 (p) and ACI10 harboring the *mcc* complementation vector ACI10 (p*mcc*) against DH5α WT and DH5α-R used for quantification analysis for Figure 3B.

(B) Proximity spotting assay of ACI15*,* ACI15 harboring the empty vector ACI15 (p) and ACI15 harboring the *mcc* complementation vector ACI15 (p*mcc*) against DH5α WT (WT) and DH5α-R (R) used for quantification analysis for Figure 3B.

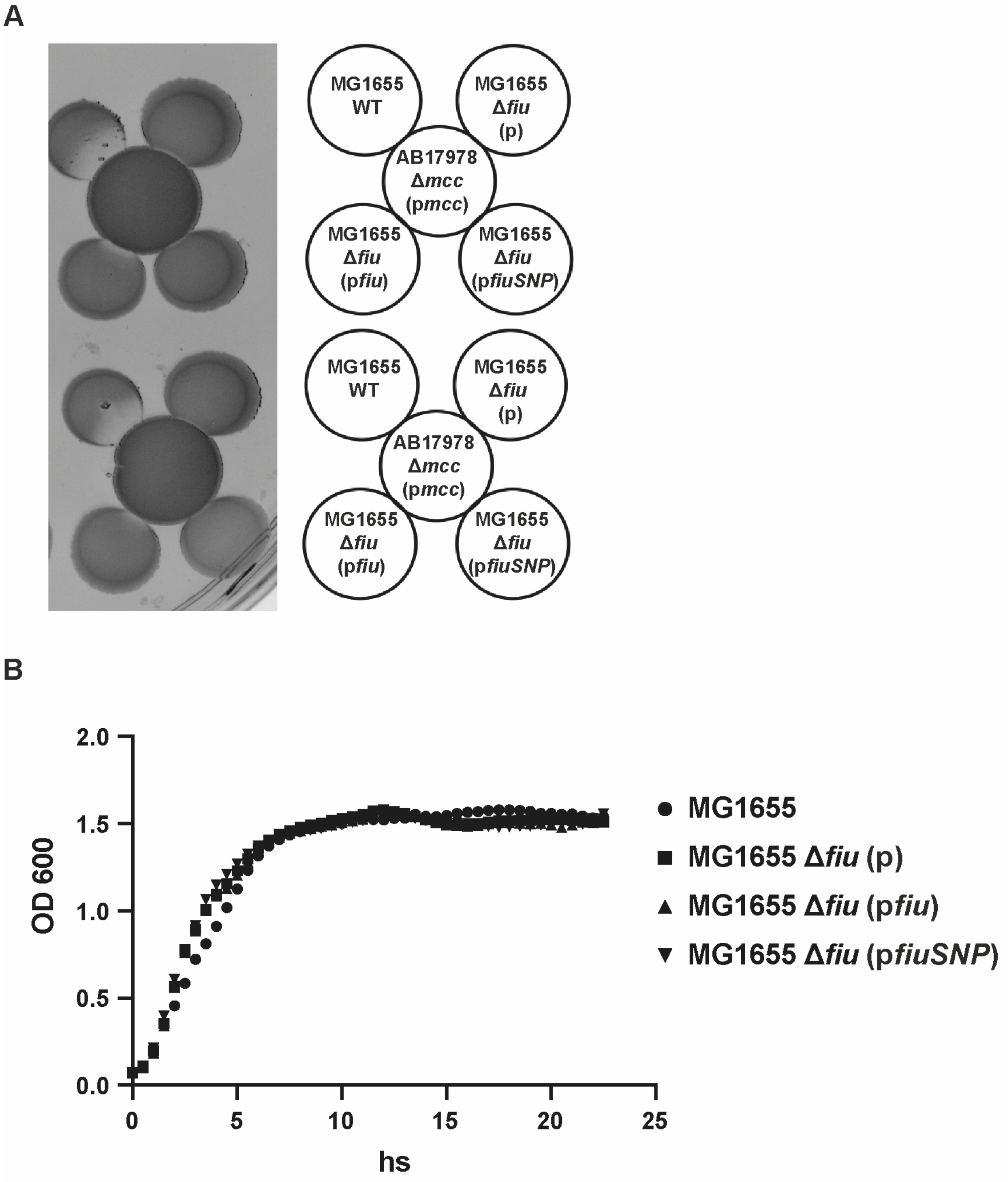

FIG S5 Fiu is the target of microcin system.
(A) Biological duplicates of proximity spotting assay using Δ*mcc* (p*mcc)* against *E. coli* MG1655, MG1655 Δ*fiu* harboring the empty vector Δ*fiu* (p), MG1655 Δ*fiu* harboring a complementing plasmid with wild-type *fiu* Δfiu (p*fiu*), and MG1655 Δ*fiu* harboring the *fiu Y657D* allele Δ*fiu* (p*fiuSNP*). Expression of the *fiu* alleles was induced with 0.01% arabinose.
(B) Growth curves of MG1655 WT, Δ*fiu* (p), Δfiu (p*fiu*), and Δfiu (p*fiuSNP*).

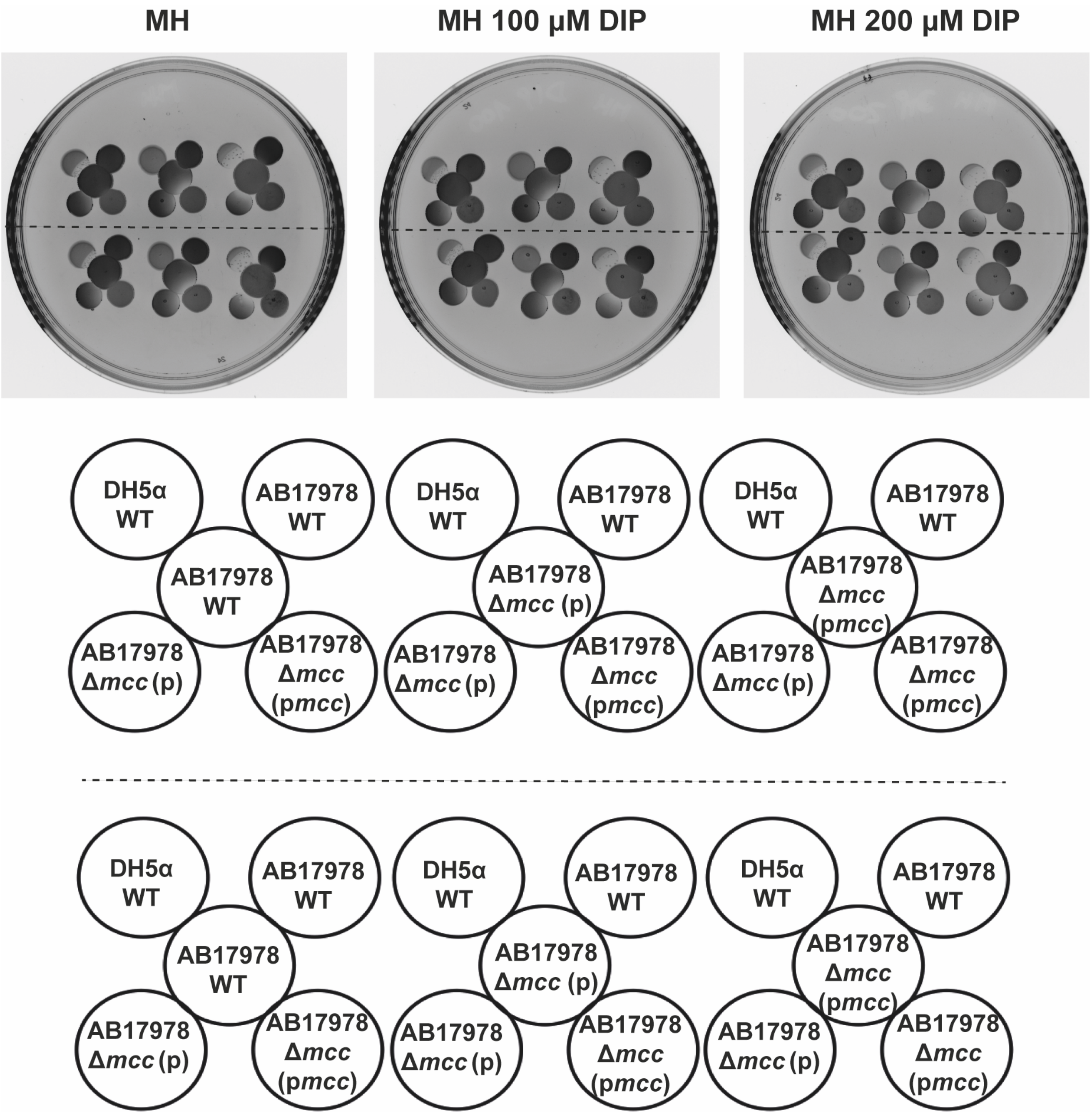

FIG S6 Iron levels regulate the *mcc* locus in AB17978. Proximity spotting assay using AB17978 WT, Δ*mcc*, and Δ*mcc* (p*mcc*) against DH5α, AB17978 WT, Δ*mcc*, and Δ*mcc* (p*mcc*) in MH media (left panel), in MH media supplemented with 100 µM DIP (middle panel) 200 µM DIP (right panel). Biological and technical duplicates used for quantification analysis in Figure 5C.

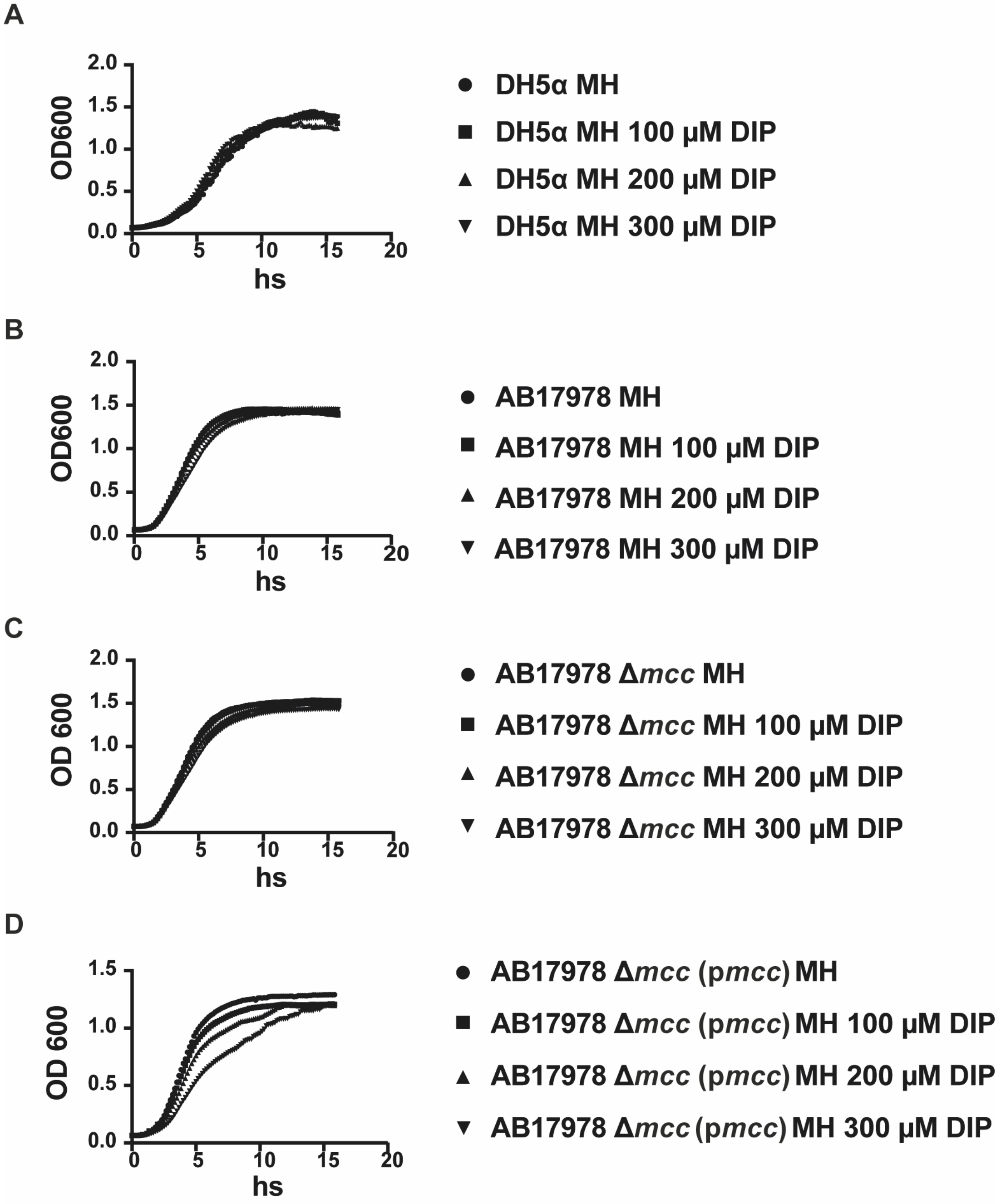

FIG S7 Growth curves of DH5α, AB17978 WT, Δ*mcc*, and Δ*mcc* (p*mcc*) in MH and MH complemented with different concentrations of DIP.

(A) Growth curves of DH5α grown in MH containing 0 µM, 100 µM, 200 µM, and 300 µM DIP.

(B) Growth curves of AB17978 WT grown in MH containing 0 µM, 100 µM, 200 µM, and 300 µM DIP.

(C) Growth curves of Δ*mcc* grown in MH containing 0 µM, 100 µM, 200 µM, and 300 µM DIP.

(D) Growth curves of Δ*mcc* (p*mcc*) grown in MH containing 0 µM, 100 µM, 200 µM, and 300 µM DIP.

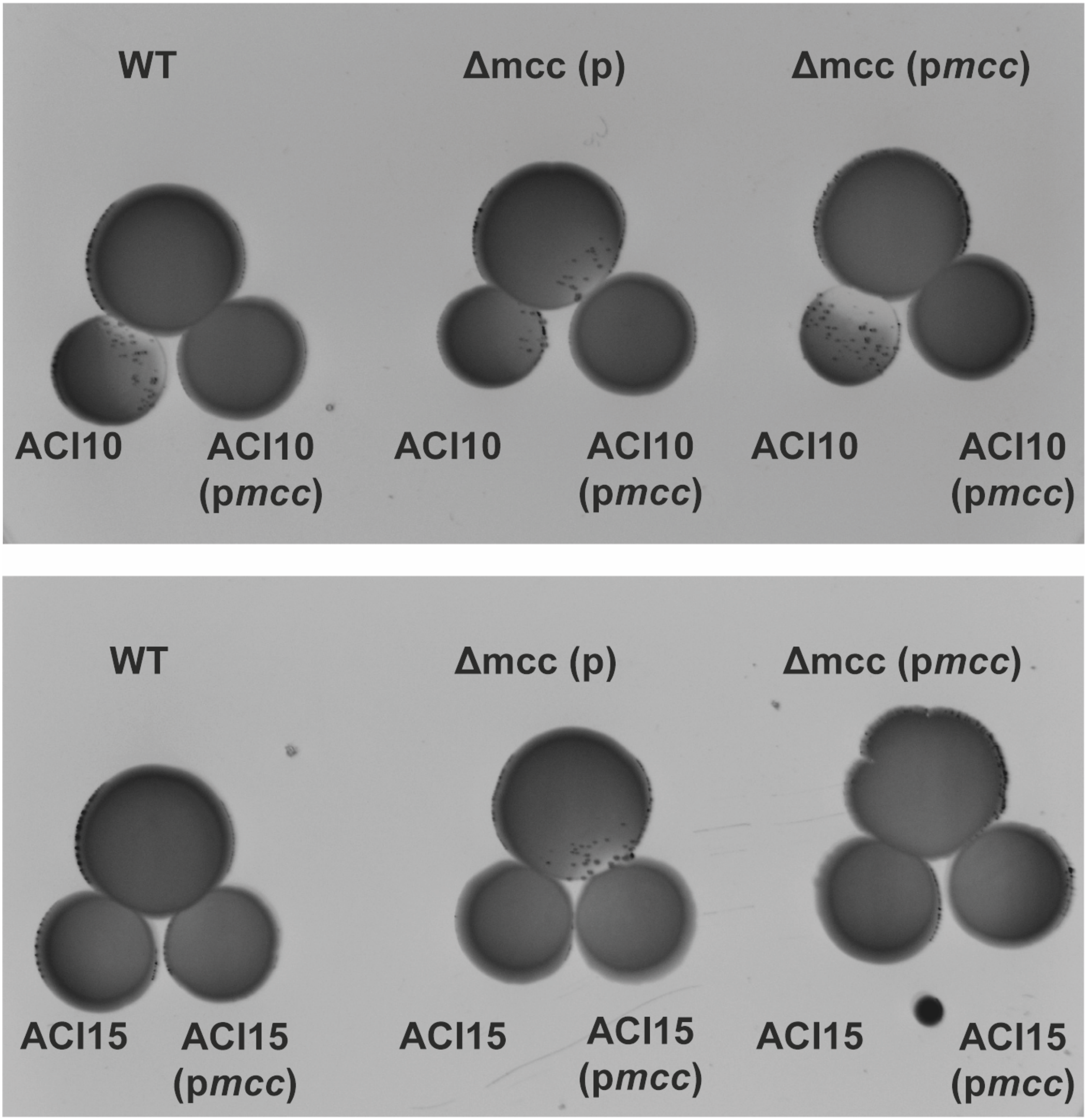

FIG S8 AB17978 inhibits *Acinetobacter pittii* ACI10, and *Acinetobacter baumannii* ACI15 is protected from AB17978 antimicrobial activity. Proximity spotting assay of AB17978 WT, Δ*mcc* (p), and Δ*mcc* (p*mcc*) against ACI10 (p) and ACI10 (p*mcc*) (top panel), and ACI15 (p) and ACI15 (p*mcc*) (bottom panel).

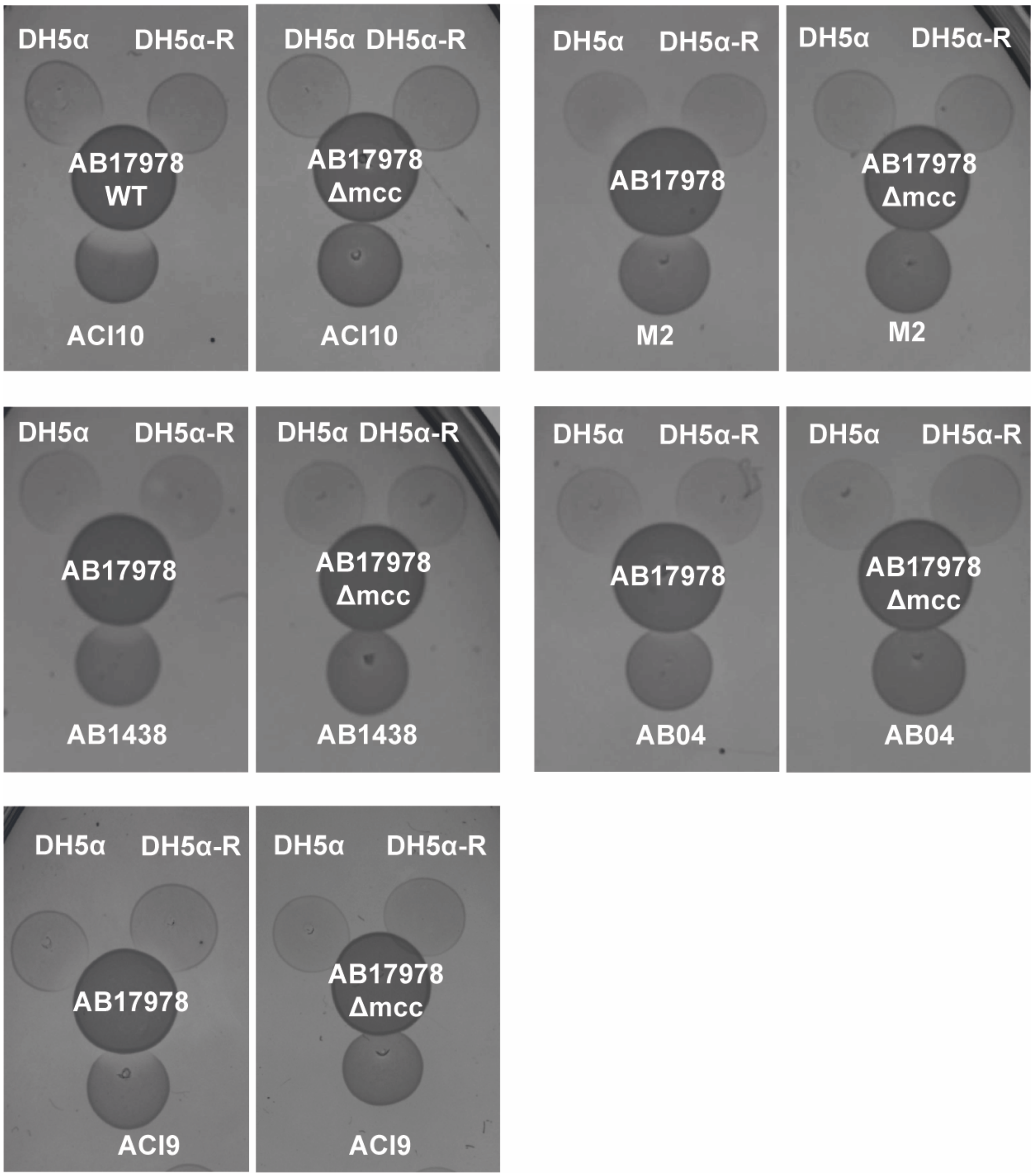

FIG S9. *Acinetobacter* strains inhibited by AB17978. Proximity spotting assay of AB17978 and Δ*mcc* against ACI10, AB1438, ACI9, M2, AB04.

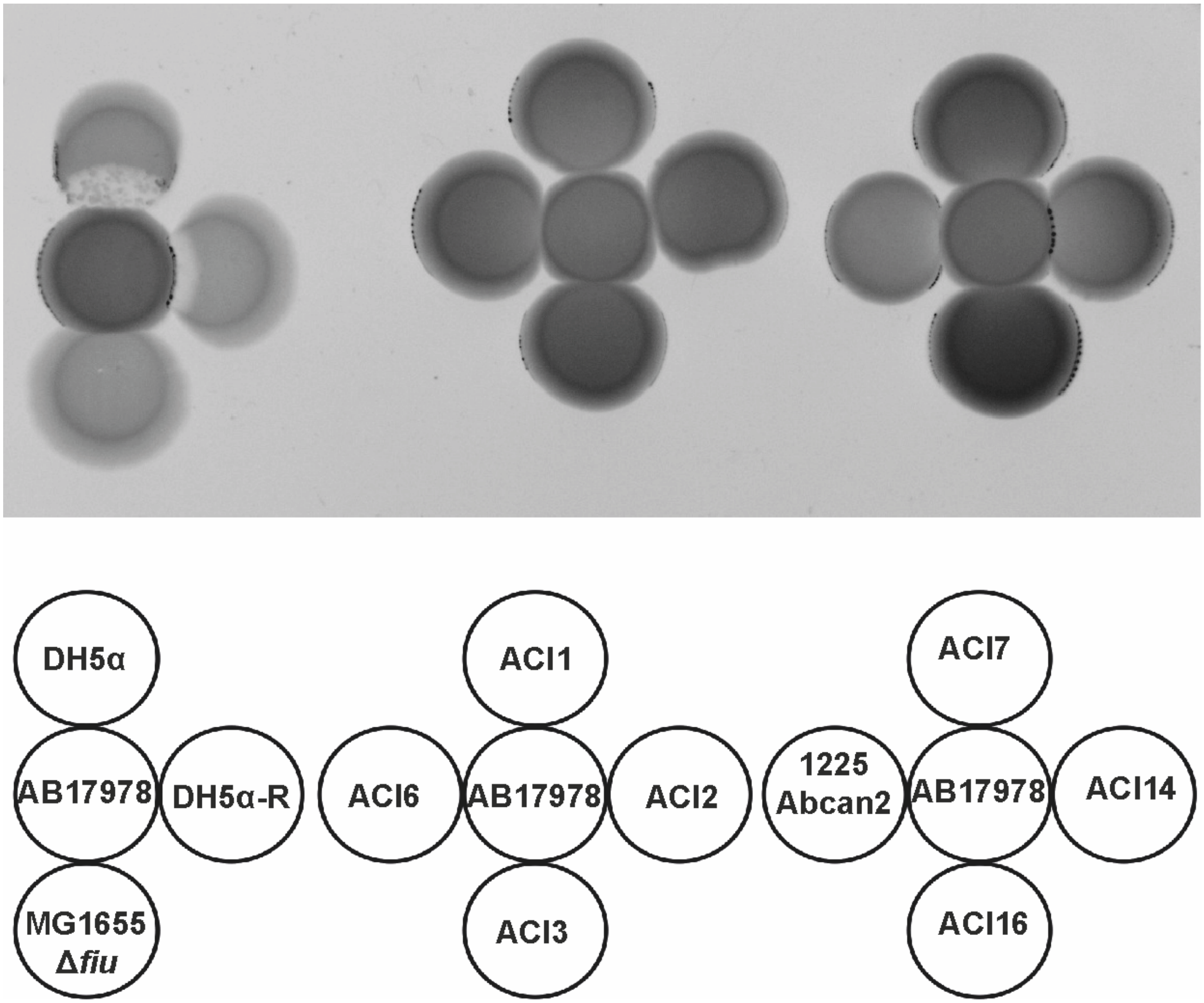

FIG S10. *Acinetobacter* strains resistant to AB17978. Proximity spotting assay of AB17978 and ACI1, ACI2, ACI3, ACI6, ACI7, ACI14, ACI16 and 1225 Abcan2, using *E. coli* DH5α, DH5α-R and MG1655 Δ*fiu* as controls.

Supplementary Table S1		
**Strains**		
Acinetobacter	**Description**	**Source**
**AB17978 pAB3+**	A. *baumannii* ATCC 17978 with pAB3 plasmid S/T resistant NCBI accession number CP012004.1	1
**AB17978 pAB3-**	*A. baumannii* ATCC 17978 without pAB3 plasmid S/T sensitive	Mario Feldman
**AB17978 ΔtssM**	*A. baumannii* ATCC 17978 T6SS mutant strain	2
**Δ*mcc***	AB17978 Δ*ACX60_RS04750-ACX60_RS04770*	This study
**Δ*mcc* (p)**	AB17978 Δ*ACX60_RS04750-ACX60_RS04770* carrying pVRL2	This study
**Δ*mcc* (p*mcc*)**	AB17978 Δ*ACX60_RS04750-ACX60_RS04770* carrying pVRL2-*ACX60_RS04750-ACX60_RS04770*	This study
**ACI10**	Clinical Isolate from surveillance swab	Lance Peterson
**ACI10 (p)**	ACI10 carrying pVRL2	This study
**ACI10 (p*mcc*)**	ACI10 carrying pVRL2-*ACX60_RS04750-ACX60_RS04770*	This study
**ACI15**	Clinical Isolate from sepsis wound	Lance Peterson
**ACI15 (p)**	ACI15 carrying pVRL2	This study
**ACI15 (p*mcc*)**	ACI15 carrying pVRL2-*ACX60_RS04750-ACX60_RS04770*	This study
**ACI1**	*A. baumannii,* clinical Isolate from blood, 2004 outbreak, MDR	Lance Peterson
**ACI2**	*A. baumannii,* clinical Isolate from blood, MDR	Lance Peterson
**ACI3**	*A. baumannii,* clinical Isolate from Jackson Pratt drain, MDR	Lance Peterson
**ACI6**	*A. baumannii,* clinical Isolate from blood, MDR	Lance Peterson
**ACI7**	*A. baumannii,* clinical Isolate from urine, MDR	Lance Peterson
**ACI9**	*A. baumannii,* clinical Isolate from blood venipuncture	Lance Peterson
**ACI14**	*A. baumannii,* clinical Isolate possibly from wound	Lance Peterson
**ACI16**	*A. baumannii,* clinical Isolate from peritoneal fluid	Lance Peterson
**AB1438**	Clinical isolated Ab1438 containing resistant plasmid, type VI secretion system (T6SS) inactive	1
**AB04**	AB04 containing pBA04, antibiotic resistant, T6SS inactive	1
**1225 abcan2**	Clinical isolate from Coccyx	1
**M2**	Clinical isolate *A. nosocomialis*	5

Supplementary Table S1		
**M2 Δ*piuA***	M2 lacking *piuA*	This study

strains		
Escherichia coli	**Description**	**Source**
**DH5α**	F– *endA1 glnV44 thi-1 recA1 relA1 gyrA96 deoR nupG purB20 φ80dlacZΔM15 Δ(lacZYA-argF)U169, hsdR17(rK–mK+), λ–*	Invitrogen
**DH5α-R**	DH5α carrying *fiu* T1969G	This study
**MG1655**	F– *λ– ilvG– rfb-50 rph-1*	Raivio Laboratory (University of Alberta)
**Δ*fiu***	MG1655 *Δfiu*	This study
**Δ*fiu* (p)**	MG1655 *Δfiu* carrying pBAD24	This study
**Δ*fiu* (p*fiu*)**	MG1655 *Δfiu* carrying p*fiu*	This study
**Δ*fiu* (p*fiuSNP*)**	MG1655 *Δfiu* carrying p*fiuSNP*	This study
**BL21**	F– *ompT gal dcm lon hsdSB(rB–mB–) λ(DE3 [lacI lacUV5-T7p07 ind1 sam7 nin5]) [malB+]K-12(λS) pLysS[T7p20 orip15A]*(CmR)	Invitrogen
**BW25113**	*lacI+rrnBT14 ΔlacZWJ16 hsdR514 ΔaraBADAH33 ΔrhaBADLD78 rph-1*<br>*Δ(araB–D)567 Δ(rhaD–B)568 ΔlacZ4787(::rrnB-3) hsdR514 rph-1*	6

Plasmids	Description	Source
**pVRL2**	pVRL1b carrying the araC-PBAD arabinose-inducible expression cassette; Gmr	7
**p*mcc***	pVRL2 carrying microcin locus (*ACX60_RS04750-ACX60_RS04770*)	This study
**p*mcaBCD***	pVRL2 carrying ACX60_RS04750-ACX60_RS04760 with endogenous promoter	This study
**p*mcaB***	pVRL2 carrying cupin (ACX60_RS04760) with endogenous promoter	This study
**pBAD24**	pBAD vector, pBR322 ori, araC, AmpR	8
**p*fiu***	pBAD24 carrying *fiu*	This study
**p*fiuSNP***	pBAD24 carrying *fiu* T1969G	This study
**pKD4**	KanR, carrying Kanamycin cassette	9, 10
**pAT03**	pMMB67EH with FLP recombinase	9
**pAT04**	pMMB67EH with RecAbsystem, TetR	9
**pCP20**	FLP recombinase	10
**pKD46**	Lambda Red recombinase expression plasmid, AmpR	10
**p*mcc-luxAB***	pHK001 carrying *mcc17978-luxAB*	This study
**pEX18-Ap_UD_piuA_M2**	pEX18-Ap carrying 1,000 bp upstream and 900 bp downstream *piuA* from M2, ApR	This study

Supplementary table S2	
**Primers**	**Sequence**
**F_U_*mcc*_500bp_KO**	CTTGGAATGATCTGTCAGAAAGTACAC
**R_U_*mcc*_500bp_KO**	TCCAGCCTACACAATCGCAGCTTTAACTTTGAAGGTGTGAGC
**F_D_*mcc*_600bp_KO**	TAAGGAGGATATTCATATGTAATCTTTTTAATAACTTAGATTAGCATTTGAAGTACAACAC
**R_D_*mcc*_600bp_KO**	TGTTGTGATTGCAGGACTATTGG
**R_*mcc*_pVRL2**	CCGCTCTAGAACTAGTGGATCCCCCGGGCTAAAGCATTTGATCACTCACAATTTG
**P1**	GCGATTGTGTAGGCTGGA
**P2**	CATATGAATATCCTCCTTA
**F_*mcc*_pVRL2**	CTTGGAATGATCTGTCAGAAAGTACAC
**R_*mcc*_pVRL2**	CCGCTCTAGAACTAGTGGATCCCCCGGGCTAAAGCATTTGATCACTCACAATTTG
**F_pVRL2_backbone**	CGAGGTCGACGGTATCG
**R_pVRL2_backbone**	ACTGGCCGTCGTTTTAC
**F_*mcc*-luxAB**	GGGGGTACCTTTATGAAAGAAGTCTATTATTGACATGG
**R_*mcc*-luxAB**	GGGTCTAGAACAAATACCCAATAGATTATAAAAAATACAATTAATATATGAATC
***fiu*F1**	CCTGACGCGCAACGGC
***fiu*F2**	ACCGCAAAAAGCCAACAC
***fiu*R1**	AGTCGAGATTGCGATTAAC
**F_*fiu*_KO**	AACATATAAGAAAAAGTCACCTGCAAAATGGTGTAGGCTGGAGCTGCTTC
**R_*fiu*_KO**	AAGTGGGGCCTGCGCCCCACATCTGAATCACATATGAATATCCTCCTTAG
**F_*fiu*_KO_confirmation**	CAATGGGTGATAGAAAATCGCTCC
**R_*fiu*_KO_confirmation**	GCAGGGCGGCGTATAACG
**F_p*fiu***	AGGAATTCACCATGGTACCCGGGATGGAAAACAATCGCAATTTC
**R_p*fiu***	GTCGACTCTAGAGGATCCCCGGGTCAGAAATGCATATTGGC

Supplementary table S2	
**F_pBAD24_backbone**	ATGCCATAGCATTTTTATCC
**R_pBAD24_backbone**	GATTTAATCTGTATCAGG
**F_*mcaBCD*_pVRL2**	GCTTGATATCGAATTCCTGCAGCCCGGGAAATTGAGGAGTAGTTTTTATGTC
**R_*mcaBCD*_pVRL2**	CCGCTCTAGAACTAGTGGATCCCCCGGGCTAAAGCATTTGATCACTCAC
**F_*mcaB*_pVRL2**	GCTTGATATCGAATTCCTGCAGCCCGGGAAATTGAGGAGTAGTTTTTATGTC
**R_*mcaB*_pVRL2**	CCGCTCTAGAACTAGTGGATCCCCCGGGCTAAAGCATTTGATCACTCAC
**F_U_*piuA*_M2_1000bp**	TGCATGCCTGCAGGTCGACTCTAGAATCATACGACCTGAACCTAAC
**F_R_*piuA*_M2_1000bp**	GACTAACTTACATGACTTTCAGGACTTAAATACAAAAC
**F_D_*piuA*_M2_900bp**	GAAAGTCATGTAAGTTAGTCTAGAATCCCATAATCTTTTTATG
**F_D_*piuA*_M2_900bp**	GCTCGGTACCCGGGGATCCTCTAGATTTTCTAATTTTGCGCCGTTTAATG
**F_*piuA*_M2_check**	CGCATAACGTTTTGTATTTAAGTCC
**R_*piuA*_M2_check**	GTTACTTTGCCATTGACCCATTC

Supplementary Material and Methods

Endogenous expression and secretion of Mcc17978.

AB17978 WT, Δ*mcc* and Δ*mcc (*p*mcc)* were grown overnight in 50 ml of MH media containing 200 µM DIP. Bacterial cells were harvested by centrifugation. Culture supernatants were precipitated overnight at 4 °C with 15% (V/V) trichloroacetic acid (TCA) and washed three times with 100% cold acetone. All samples were resuspended in 200 mM HEPES pH 8 and SDS-PAGE sample buffer. Whole-cell and culture supernatant samples were separated by SDS-PAGE and stained with simplyBlue. Protein bands were imaged with the LICOR Odyssey scanner and analyzed with the Image studio 5.0 software.

Nucleotide alignment

Genomic sequences of AB17978, ABD36, ABVB2486, ABPK1, AB5457 and ABCTX13 were extracted from NCBI, and nucleotide alignment of Tn6171 and microcin locus the mentioned strains were performed using Geneious Prime 2021.2.2.

Protein sequence alignment

Protein sequence alignments of microcins AKQ26059.1 (AB17978), ALJ86107.1 (D36), ATR85905.1 (ABPK1), QRN20889.1 (ABCTX13) and QJH01317.1 (ABVB2486) were performed using Global alignment with free end gaps with Geneious Prime 2021.2.2.

Signal peptide prediction

The signal peptide of microcin was predicted using SignalP 6.0 tool (11).

Supplementary references

1. Weber BS, Ly PM, Irwin JN, Pukatzki S, Feldman MF. 2015. A multidrug resistance plasmid contains the molecular switch for type VI secretion in *Acinetobacter baumannii*. Proc Natl Acad Sci U S A 112:9442-7.

2. Weber BS, Miyata ST, Iwashkiw JA, Mortensen BL, Skaar EP, Pukatzki S, Feldman MF. 2013. Genomic and functional analysis of the type VI secretion system in *Acinetobacter*. PLoS One 8:e55142.

3. Tilley D, Law R, Warren S, Samis JA, Kumar A. 2014. CpaA a novel protease from *Acinetobacter baumannii* clinical isolates deregulates blood coagulation. FEMS Microbiol Lett 356:53-61.

4. Di Venanzio G, Flores-Mireles AL, Calix JJ, Haurat MF, Scott NE, Palmer LD, Potter RF, Hibbing ME, Friedman L, Wang B, Dantas G, Skaar EP, Hultgren SJ, Feldman MF. 2019. Urinary tract colonization is enhanced by a plasmid that regulates uropathogenic *Acinetobacter baumannii* chromosomal genes. Nat Commun 10:2763.

5. Carruthers MD, Nicholson PA, Tracy EN, Munson RS, Jr. 2013. *Acinetobacter baumannii* utilizes a type VI secretion system for bacterial competition. PLoS One 8:e59388.

6. Baba T, Ara T, Hasegawa M, Takai Y, Okumura Y, Baba M, Datsenko KA, Tomita M, Wanner BL, Mori H. 2006. Construction of *Escherichia coli* K-12 in-frame, single-gene knockout mutants: the Keio collection. Mol Syst Biol 2:2006 0008.

7. Lucidi M, Runci F, Rampioni G, Frangipani E, Leoni L, Visca P. 2018. New Shuttle Vectors for Gene Cloning and Expression in Multidrug-Resistant *Acinetobacter* Species. Antimicrob Agents Chemother 62.

8. Guzman LM, Belin D, Carson MJ, Beckwith J. 1995. Tight regulation, modulation, and high-level expression by vectors containing the arabinose PBAD promoter. J Bacteriol 177:4121-30.

9. Tucker AT, Nowicki EM, Boll JM, Knauf GA, Burdis NC, Trent MS, Davies BW. 2014. Defining gene-phenotype relationships in *Acinetobacter baumannii* through one-step chromosomal gene inactivation. mBio 5:e01313-14.

10. Datsenko KA, Wanner BL. 2000. One-step inactivation of chromosomal genes in *Escherichia coli* K-12 using PCR products. Proc Natl Acad Sci U S A 97:6640-5.

11. Teufel F, Almagro Armenteros JJ, Johansen AR, Gislason MH, Pihl SI, Tsirigos KD, Winther O, Brunak S, von Heijne G, Nielsen H. 2022. SignalP 6.0 predicts all five types of signal peptides using protein language models. Nat Biotechnol doi:10.1038/s41587-021-01156-3.
